# The Kynurenine Pathway in Tuberculosis: Focus on Immunometabolic Regulation, Compartment-Specific Biology, and Therapeutic Potential

**DOI:** 10.3390/life16081350

**Published:** 2026-08-17

**Authors:** Piotr Stasiak, Kacper Kraśnik, Maciej Biskupski, Aleksandra Tarka, Michał Flis, Ewa M. Urbańska

**Affiliations:** Department of Experimental and Clinical Pharmacology, Medical University of Lublin, Jaczewskiego 8b, 20-090 Lublin, Poland; 63423@umlub.edu.pl (P.S.); 63112@umlub.edu.pl (K.K.); maciej.biskupski@umlub.edu.pl (M.B.); 63477@umlub.edu.pl (A.T.);

**Keywords:** tuberculosis, kynurenine pathway, tryptophan metabolism, IDO1, immunometabolism, granuloma, host-directed therapy

## Abstract

Tuberculosis (TB) remains a global health problem, and attention is focused on host immunometabolic pathways influencing antimicrobial immunity, tissue injury, and treatment response. This comprehensive review integrates evidence on the kynurenine pathway (KP) of tryptophan (TRP) metabolism in TB and evaluates its potential as a host-directed therapeutic target. Evidence from human cohorts, in vitro systems, animal models, and non-human primates indicates that *Mycobacterium tuberculosis* activates the KP across myeloid, antigen-presenting, non-hematopoietic lung, granulomatous, pleural, and central nervous system compartments. KP activation is shaped by interferon-γ signaling, mycobacterial burden, infection duration, strain virulence, and host regulatory mechanisms. Increased indoleamine 2,3-dioxygenase-related (IDO) activity, TRP depletion, kynurenine accumulation, and kynurenine–aryl hydrocarbon receptor (AhR) signaling are associated with impaired T-cell proliferation and recruitment, suppressive myeloid phenotypes, and spatially restricted immunoregulatory niches within granulomas. Accumulated data indicate context-dependent protective effects of the KP manipulations, through restriction of excessive inflammation. Furthermore, the KP-related changes may have biomarker value. Integrated assessment of enzyme expression, kynurenine-to-tryptophan ratio (K/T ratio), downstream metabolites, and signaling pathways will be essential for translational studies. Future studies should therefore evaluate KP activity using integrated readouts, including IDO expression, metabolites and their ratios, and KYN–AhR signaling, and should determine whether context-specific pathway modulation can safely improve outcomes when combined with conventional anti-TB therapy.

## 1. Introduction

Tuberculosis (TB), caused by *Mycobacterium tuberculosis* (Mtb), remains one of the most severe infectious diseases worldwide despite the availability of effective antimicrobial therapy and intensified research efforts aimed to detect it early. According to the World Health Organization, TB continues to affect more than 10 million people annually and remains a leading cause of death from a single infectious agent, with approximately 1.23 million deaths reported in 2024 [1]. Although the burden is greatest in low- and middle-income countries, TB remains relevant in all regions. In the European Union/European Economic Area, 38,993 TB cases were reported in 2023, corresponding to a notification rate of 8.6 per 100,000 population [2]. The continued global impact of TB reflects not only transmission dynamics and social determinants of health, but also important biological and clinical challenges, including delayed diagnosis, human immunodeficiency virus (HIV) co-infection, drug resistance, extrapulmonary disease and incomplete treatment adherence [1,2,3,4].

Current therapeutic strategies for TB are effective in many patients but remain imperfect. Standard treatment requires prolonged multidrug regimens. Furthermore, the management of drug-resistant TB is more complex, less tolerable and associated with poorer outcomes [3,4]. Moreover, antimicrobial therapy alone does not directly address the host inflammatory and immunoregulatory processes that contribute to tissue damage, bacterial persistence and variable clinical response. These limitations have stimulated interest in host-directed therapy (HDT), which aims to modulate host immune pathways in order to enhance antimicrobial defence, reduce immunopathology and potentially improve treatment outcomes when used alongside conventional anti-TB drugs [5,6]. In this context, immunometabolic pathways have emerged as particularly relevant, because they integrate inflammatory signalling, nutrient availability, cellular activation and immune regulation [7,8].

**Figure 1:**
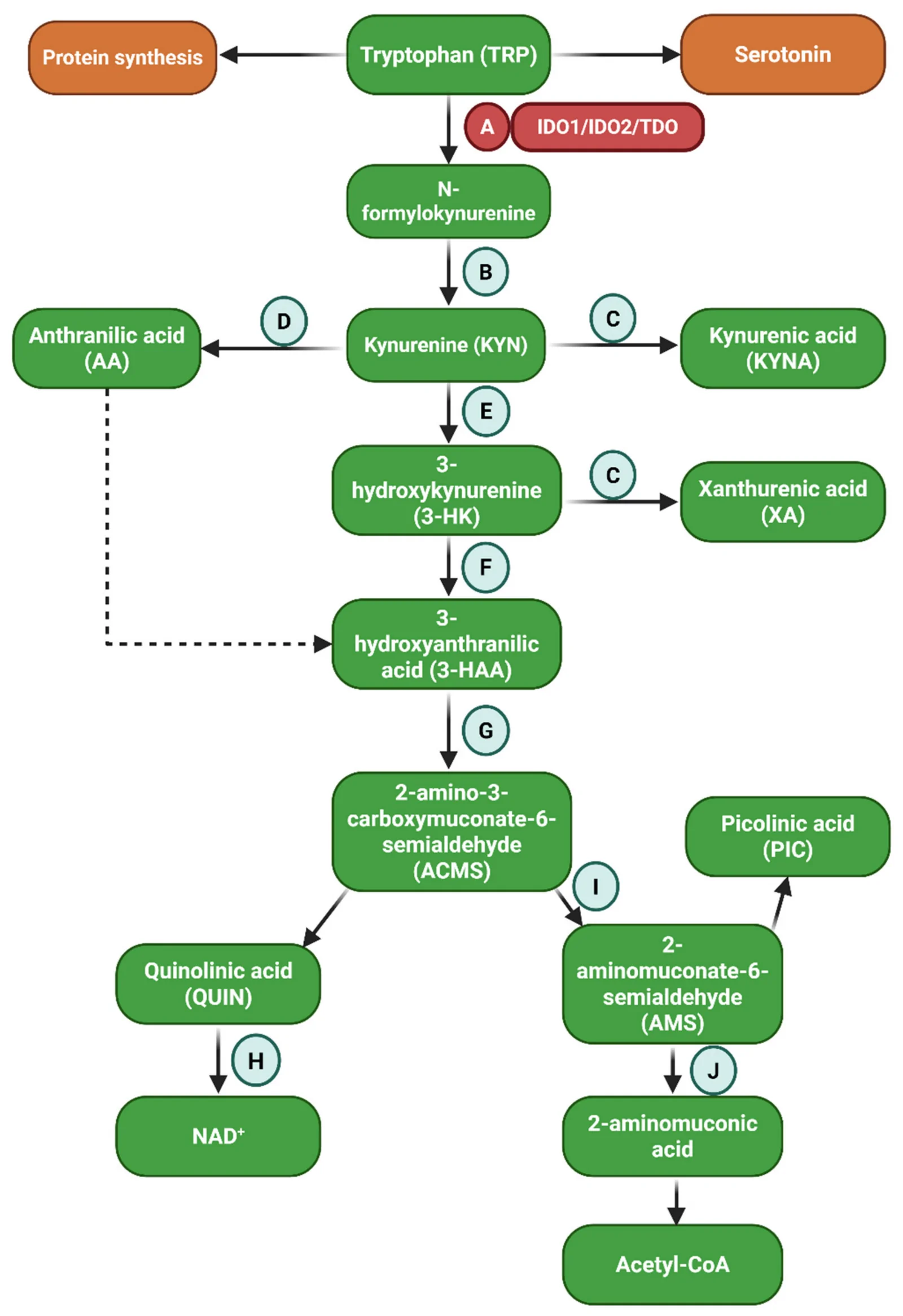
**Overview of TRP metabolism through the KP.** TRP may be utilized for serotonin and protein synthesis or metabolized through the KYN pathway (KP). The reactions are catalyzed by: (A) indoleamine 2,3-dioxygenase (IDO) 1/2, or tryptophan 2,3-dioxygenase (TDO); (B) kynurenine formamidase; (C) kynurenine aminotransferases (KAT I–IV); (D) kynureninase; (E) kynurenine 3-monooxygenase (KMO); (F) kynureninase; (G) 3-hydroxyanthranilic acid 3,4-dioxygenase; (H) quinolinate phosphoribosyltransferase; (I) 2-amino-3-carboxymuconate-6-semialdehyde decarboxylase; and (J) 2-aminomuconate-6-semialdehyde dehydrogenase. KYN may also be converted to kynurenic acid (KYNA) by KATs, whereas 3-hydroxykynurenine (3-HK) may be converted to xanthurenic acid by KATs. Anthranilic acid may be converted to 3-hydroxyanthranilic acid (3-HAA) through nonspecific hydroxylation. The pathway subsequently leads to the formation of quinolinic acid (QUIN), NAD^+^, picolinic acid, 2-aminomuconic acid, and acetyl-coenzyme A (acetyl-CoA). Dashed arrows indicate alternative or indirect metabolic routes. Created in BioRender. Stasiak, P. (2026). https://BioRender.com/aiwossg.

The kynurenine pathway (KP) is the principal route of tryptophan (TRP) degradation in mammals [9]. The principal enzymatic steps and major metabolic branches of the kynurenine pathway are summarized in Figure 1.

Step-limiting enzymes, indoleamine 2,3-dioxygenase (IDO) 1/2 or tryptophan 2,3-dioxygenase (TDO), convert TRP to kynurenine (KYN) via an intermediate product, N-formylkynurenine. IDO and TDO differ substantially in their characteristics, tissue distribution, regulation, and catalytic efficiency. Under physiological conditions, TDO is predominantly hepatic and accounts for most systemic TRP degradation, whereas extrahepatic IDO-dependent metabolism becomes more prominent during immune activation [9,10]. IDO1 is widely distributed in extrahepatic tissues and immune cells and is strongly inducible by inflammatory signals, particularly IFN-γ, whereas IDO2 has a more restricted tissue distribution, substantially lower catalytic activity toward TRP than IDO1, and less well-defined inflammatory regulation [9,10,11]. Thus, although all three enzymes can initiate TRP catabolism along the KP, their quantitative and context-dependent contributions to TRP catabolism are not equivalent.

KYN then serves as a central metabolic branching point. It may be transaminated by kynurenine aminotransferases (KATs) to kynurenic acid (KYNA), converted by kynurenine 3-monooxygenase (KMO) to 3-hydroxykynurenine, or further metabolized through downstream enzymatic steps leading to 3-hydroxyanthranilic acid (3-HAA), quinolinic acid (QUIN), picolinic acid (PIC) and nicotinamide adenine dinucleotide (NAD+) synthesis [9,10,11,12]. Thus, the KP should not be regarded as a simple linear cascade driven only by IDO activity, but rather as a branched and compartment-dependent metabolic network.

This distinction is particularly important for the interpretation of KP-related biomarkers. The K/T ratio is commonly used to assess the activity of initial TRP catabolism and as a surrogate marker of pathway activity. Although the ratio can reflect changes in IDO activity in closed in vitro systems, its indiscriminate use as a measure of IDO activity in vivo is not justified, because circulating TRP and KYN concentrations are also influenced by hepatic TDO activity, plasma free-TRP flux, and downstream KYN metabolism, including KMO and kynureninase [13]. Increased conversion of TRP to KYN may be followed by different downstream metabolic patterns depending on cell type, tissue compartment, inflammatory milieu, substrate availability, and the relative activity of KAT-, KMO-, and kynureninase-dependent branches [10,11,12,13]. Accordingly, an elevated K/T ratio does not reflect the activity of the enzyme responsible for TRP-to-KYN metabolism and should not be interpreted as a direct measure of IDO catalytic activity. Because IDO1, IDO2, and TDO2 can all catalyse the initial KP step, the K/T ratio alone cannot resolve their relative contributions [11,13]. The levels of KYNA, 3-HK, 3-HAA, and QUIN may change independently rather than proportionally. Therefore, interpretation of KP-related readouts in TB requires careful distinction between upstream TRP catabolism, downstream metabolite redistribution, and functional immune effects.

During infection, these metabolic differences acquire immunological relevance. IDO1-dependent TRP catabolism can influence immune responses through changes in TRP availability and the generation of KYN and downstream metabolites [14,15]. Resulting KP activation may influence T-cell proliferation, antigen-presenting cell (APC) function, macrophage activation, regulatory immune phenotypes, oxidative stress and aryl hydrocarbon receptor (AhR)—dependent signaling [8,14,16]. These mechanisms are potentially relevant to TB, where protective immunity depends on a finely balanced response: insufficient cellular immunity favors bacterial growth, whereas excessive or dysregulated inflammation contributes to tissue destruction, cavitation and disease progression [7,17].

Thus, in TB, the KP is of interest not only as a source of potential inflammatory markers, but also as a possible contributor to disease biology. Mtb infection develops within highly organised and heterogeneous tissue environments, including granulomas in which immune activation, bacterial survival, necrosis and cellular exclusion may coexist [17,18]. Metabolic pathways operating within these compartments may influence whether the host response promotes bacterial control, immune tolerance or tissue pathology. The KP is particularly suited to this type of investigation because it links interferon-driven inflammation with local amino-acid metabolism and immunoregulatory signaling [7,14,15]. However, the functional meaning of KP activation in TB remains complex and may vary according to disease stage, anatomical compartment, host immune status, HIV co-infection, bacterial burden and treatment phase [19].

The therapeutic relevance of the KP follows from this immunometabolic role. If excessive or compartmentalised KP activation contributes to immune suppression, impaired T-cell function or ineffective granuloma immunity, then modulation of this pathway could represent a rational HDT. Conversely, because KP activity may also participate in antimicrobial restriction and control of inflammation, non-selective inhibition could theoretically produce context-dependent risks [5,6,16]. This duality makes the KP an attractive but challenging therapeutic target. A careful synthesis of existing evidence is therefore necessary to distinguish established mechanisms from preliminary therapeutic concepts and to identify where further translational or clinical validation is required.

Despite increasing interest, evidence on the KP in TB remains highly heterogeneous, covering in vitro infection models, animal and non-human primate studies, human metabolomic analyses, tissue-based investigations, and clinical studies evaluating disease activity, diagnosis, or treatment response. These studies differ substantially in biological material, analytical methodology, investigated pathway components, and clinical context, limiting direct comparison and integrated interpretation. Therefore, this review systematically organizes the available evidence according to experimental model, biological compartment, KP-related readout or intervention, and clinical relevance, while distinguishing upstream pathway activation from downstream metabolic and immunological consequences. By integrating mechanistic, translational, and biomarker-oriented findings within a common framework, we aim to clarify the current evidence for KP involvement in TB, identify areas in which findings are consistent or context-dependent, and highlight the major limitations and knowledge gaps that should guide future research.

## 2. Materials and Methods

Current evidence concerning the role of the KP metabolites, enzymes and related signaling axes in the pathophysiology and therapeutic modulation of TB was collected through an identification and screening process recommended by PRISMA guidelines [20]. Particular attention was given to the biological interpretation of KP activation, the distinction between upstream and downstream pathway readouts, the relevance of KP activity to host immunity and granulomatous inflammation, and the potential of KP-related mechanisms as targets for HDT or treatment monitoring. Because the objective was narrative integration of mechanistic and translational evidence rather than estimation of a pooled diagnostic, prognostic or therapeutic effect size, no quantitative meta-analysis was performed. The search strategy combined terms related to TRP metabolism and the KP with terms related to TB and Mtb. The core search string was: (tryptophan OR kynuren* OR “kynurenic acid” OR hydroxykynuren* OR hydroxyanthranil* OR anthranil* OR quinolin* OR picolin* OR IDO OR IDO1 OR IDO2 OR “indoleamine 2,3-dioxygenase” OR TDO OR TDO2 OR “tryptophan 2,3-dioxygenase” OR KMO OR “kynurenine 3-monooxygenase” OR kynureninase OR KAT OR “kynurenine aminotransferase”) AND (tuberculosis OR tuberculo* OR “*Mycobacterium tuberculosis*” OR “*M. tuberculosis*”). Eligible studies included original human, animal, ex vivo or in vitro research in which at least one KP-related component was measured or mechanistically evaluated in relation to TB. Eligible KP-related parameters included TRP, KYN, KYNA, 3-hydroxykynurenine, 3-HAA, anthranilic acid (AA), QUIN, PIC, K/T ratio, IDO1, IDO2, TDO/TDO2, KMO, kynureninase, KAT enzymes and downstream signalling pathways such as KYN–AhR. Studies were considered relevant if they addressed TB pathophysiology, cellular immune regulation, granuloma biology, extrapulmonary TB, HIV/TB co-infection, TB severity, treatment response, or experimental HDT. Reviews, editorials, commentaries, conference abstracts without sufficient full-text data, purely methodological papers without TB-relevant biological findings, and studies not involving TB or Mtb-relevant models were excluded from the primary synthesis, although selected reviews were used to support background information on KP biology.

Data were extracted on authorship, year of publication, study design, biological model, study population or experimental system, TB phenotype, comparator group, biological material analysed, analytical method, KP-related parameters, principal findings, therapeutic or mechanistic relevance and reported limitations. For human studies, extracted variables included clinical phenotype, HIV status where available, sample type, diagnostic reference standard and treatment status. For animal and in vitro studies, extracted variables included species or cell type, infecting mycobacterial strain, intervention, timing of sampling and immunological or microbiological outcomes. Particular attention was paid to whether a study measured direct enzyme expression, metabolite concentrations, K/T ratio or downstream signalling activity, because these readouts are biologically related but not interchangeable. Throughout this review, IDO1 and IDO2 are used when the corresponding isoenzyme was measured specifically. The term IDO is retained when the original assay or pharmacologic intervention did not distinguish between isoforms. IDO-related activity is used for indirect functional readouts, particularly the K/T ratio, when the relative contributions of IDO1, IDO2, and TDO2 cannot be resolved. Pharmacologic IDO inhibition in the studies reviewed here was performed using methyltryptophan-based compounds, including D-1-methyltryptophan (D1-MT) and racemic 1-methyl-DL-tryptophan (1-MT-DL), with some studies reporting the compound more generally as 1-MT. Because these compounds are not uniformly isoform-selective, their effects are interpreted as pharmacologic perturbation of IDO-related TRP catabolism rather than definitive evidence of selective IDO1 inhibition.

**Figure 2:**
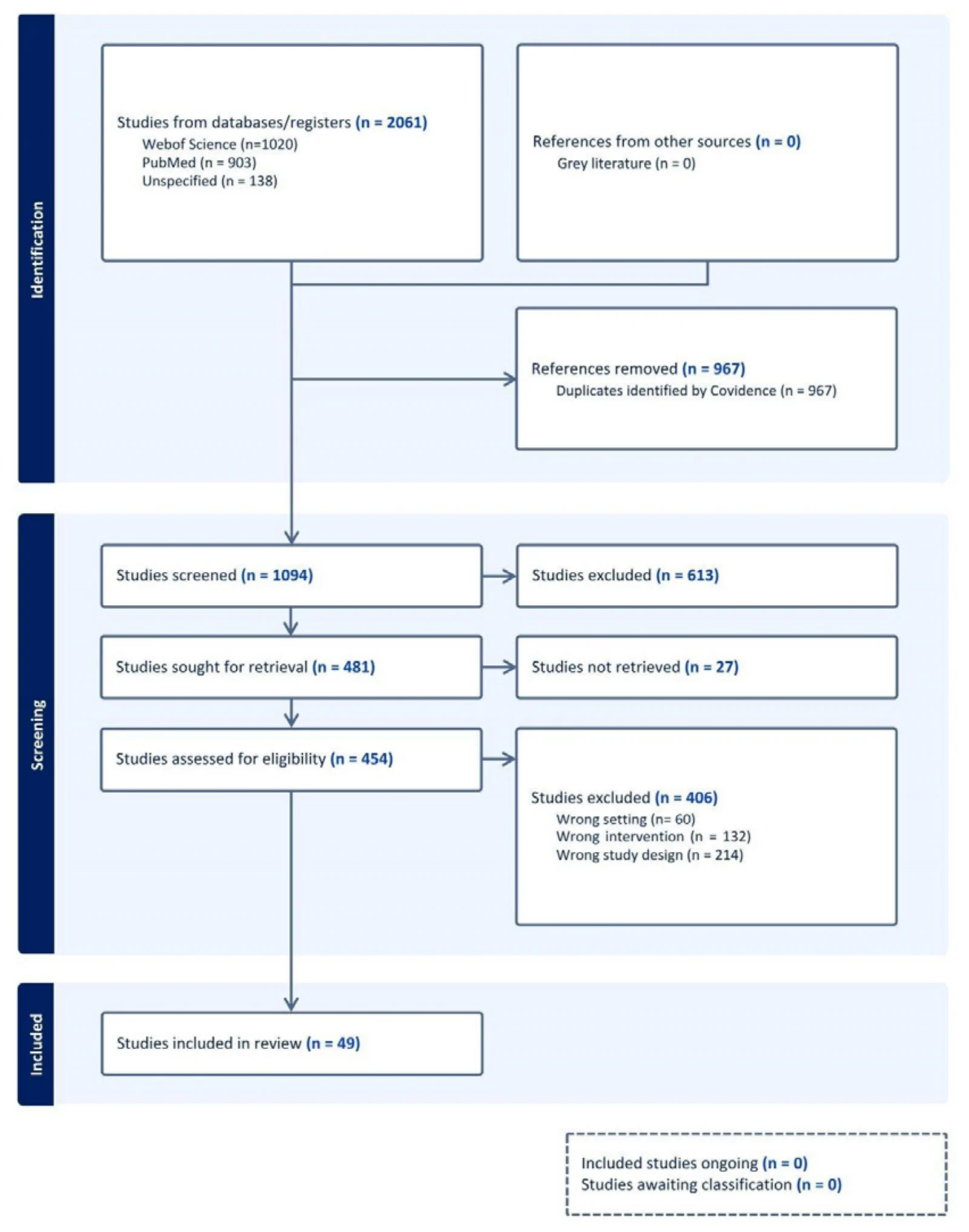
**Diagram of study identification and selection**.

The synthesis was qualitative and organised around major biological and translational themes: cellular sources of KP activity; regulation by Mtb infection, IFN-γ, pathogen burden and virulence; effects on macrophage, monocyte, dendritic-cell and T-cell function; spatial organisation of KP activity within granulomas; systemic and compartment-specific metabolite changes in pulmonary (PTB) and extrapulmonary TB; effects of HIV co-infection; treatment-associated changes in TRP catabolism; and therapeutic strategies involving IDO inhibition, KYN–AhR signalling, MET receptor tyrosine kinase (MET) modulation or broader immunometabolic interventions. Given the substantial heterogeneity in study design, host species, clinical population, biospecimen type, analytical platform and outcome definition, no pooled effect estimates were calculated. Instead, evidence was interpreted according to biological directness, consistency across models and translational relevance to human TB. The overall screening process is summarized in Figure 2.

## 3. The Kynurenine Pathway in TB

The KP operates predominantly intracellularly and can be initiated by IDO1, IDO2, or TDO2, with the relative contribution of each enzyme depending on tissue and inflammatory context [21,22]. It is well recognized that the KP has a broad cellular and tissue distribution rather than being restricted to one specialized immune-cell population. KP-related enzymes and metabolites have been described in immune cells, epithelial compartments, and nervous-system tissues [23,24,25,26]. The TB literature reviewed below is broadly consistent with this wider biological pattern, with KP activity detected across myeloid, antigen-presenting, non-hematopoietic, granulomatous, and central nervous system (CNS)—associated compartments. A compartment-specific synthesis of the principal human and animal findings is presented in Table 1 and Table 2, respectively.

### 3.1. Cellular and Tissue Sources of KP Activity

KP activity in TB is not restricted to a single immune-cell population. Pathway activity has been detected mainly in macrophages, monocytes, dendritic cells (DCs), epithelial cells, and endothelial cells, including laboratory-derived cell models [14,27,30,33,36,54,55,56,57,60,67,68,71,72]. Moreover, in tuberculous meningitis (TBM), KP activity has been detected in the parenchyma, choroid plexus, inferior olive, and cerebellum, particularly in Purkinje cells and the granular layer [48].

#### 3.1.1. Macrophages

In TB, macrophages are one of the cellular sources of kynurenines. In human material, IDO1 protein has been detected in macrophages by histopathological examination [18,44], while macrophage-based human cell models further support infection-associated pathway activation. Infected macrophages consistently showed increased IDO-related activity, as well as higher TDO2 expression [57]. Additional findings showed increased QUIN concentrations in the supernatants of infected macrophages [14]. A human monocyte-derived cell model further showed that 1,25(OH)_2_D_3_-polarized cells had lower IDO expression, which remained lower after Mtb infection compared with other cell subsets, indicating that the active form of vitamin D could have an impact on immunity in TB via KP [54].

Similarly, animal models also support the involvement of macrophages in TRP metabolism. In macaque tuberculous granulomas, macrophages show substantial IDO1 expression, supporting their role as an important cellular source of KP activity in granulomatous lesions [61,65,66]. Murine infection models further support macrophage-associated IDO1 expression and lung-associated IDO1 protein levels during TB [30,33,67], while complementary murine in vitro macrophage systems indicate that this response can be reproduced under controlled experimental conditions [14,33,55].

#### 3.1.2. Monocytes

Human monocytes show increased IDO1 expression [36,60] and abundance [27] during TB-related immune activation [27,36,60]. Monocytes are a major inducible source of IDO1 and therefore a key cellular driver of KP activation in inflammatory settings [73]. In human peripheral blood mononuclear cells (PBMCs), cluster of differentiation (CD)14^+^ monocytes show strong antigen- and IFN-γ–dependent IDO1 expression, while higher baseline systemic IDO-related activity (reflected by elevated K/T ratio) is associated with weaker antigen-specific IFN-γ responses after vaccination, consistent with an immunoregulatory role of the pre-existing KP [36].

During mycobacterial stimulation and active TB, IDO1 expression is localized to CD14^+^ monocytes and can restrain antimicrobial and activation-related monocyte programs. Pharmacologic IDO inhibition increases Bacillus Calmette–Guérin (BCG) strain expressing green fluorescent protein (GFP-BCG) uptake by CD14^+^ monocytes and enhances the expression of maturation and co-stimulatory markers, including CD40, CD83, and CD86. Moreover, mesenchymal–epithelial transition factor (MET) signaling promotes monocyte IDO1 expression and reduces mycobacterial uptake [60]. In parallel, monocytic myeloid-derived suppressor cells (M-MDSCs) expand with TB severity and exhibit activity of IDO1 in vivo, reinforcing that monocyte-lineage suppressor states can use IDO1-associated mechanisms to shape host immunity [27].

#### 3.1.3. Dendritic, Epithelial and Endothelial Cells

Human in vitro data indicate that dendritic cells (DCs) follow a pattern similar to macrophages and monocytes, with increased IDO-related activity (measured as K/T ratio) [30] and overall increased KP activity (measured as increased QUIN abundance) [14] during mycobacterial immune stimulation. Histopathological evidence from human tuberculous granulomas further confirmed IDO1 protein presence in DCs [18]. Similar IDO-related findings (like higher expression [14] and protein levels [71,72]) have been demonstrated in murine in vitro models, supporting the role of DCs as an additional source of KP activity during mycobacterial immune responses [14,71,72].

In animal models, during chronic Mtb infection, lung IDO1 expression is mainly found in non-hematopoietic parenchymal cells, with airway epithelial cells and vascular endothelial cells serving as the principal in vivo sources of IDO1 protein during this phase [68].

Taken together, the available evidence indicates that KP activation in TB is distributed across both immune and non-hematopoietic compartments rather than being attributable to a single cellular source. Evidence is most extensive for myeloid cells, particularly macrophages and monocytes, whereas the contribution of epithelial, endothelial, and other tissue-associated populations is supported by a more limited and context-specific set of studies. Importantly, much of the available source-mapping evidence is based on enzyme expression or protein localization rather than direct measurement of metabolic flux; therefore, detection of IDO1 within a given cell population does not necessarily establish its quantitative contribution to local or systemic KP metabolite production.

### 3.2. Inducers and Modulators of the KP Function in TB

KP activation in TB appears to be regulated by several converging factors, including IFN-γ signaling, mycobacterial burden, infection duration, strain virulence, and additional molecular regulators that influence IDO expression or stability.

#### 3.2.1. IFN-γ-Driven Induction

Vaccination with MVA85A, a modified vaccinia virus expressing antigen 85A from Mtb, evoked an IFN-γ-dependent increase in IDO1 expression [36]. In an uninfected human monocyte-derived cell polarization model using granulocyte–macrophage colony-stimulating factor (GM-CSF), IFN-γ, and Mtb-derived lipopolysaccharide (LPS), IDO expression was also induced [54]. In human bronchoalveolar lavage (BAL) cells and human monocyte-derived macrophages, IFN-γ stimulation and mycobacterial infection increased QUIN concentrations in culture supernatants. Interestingly, monocyte-derived macrophages produced substantially more QUIN than alveolar macrophages, suggesting that downstream KP metabolism is cell-type dependent. However, direct comparison is limited because the models differed in experimental conditions and infecting mycobacterial species [14]. In Mtb-infected bone marrow chimeric mice with IFN-γ-unresponsive non-hematopoietic cells, both lung IDO1 mRNA expression and protein levels were reduced by approximately 95%.

These results indicate that IFN-γ signaling in parenchymal non-hematopoietic compartments is a dominant driver of pulmonary IDO1 expression during chronic TB [68]. Moreover, in murine bone marrow-derived macrophages, mycobacterial infection and IFN-γ stimulation induced IDO1 expression, and combined conditions (including IFN-γ pretreatment) robustly induced IDO1 in vitro [14].

#### 3.2.2. Mycobacterial Burden, Duration of Infection, Virulence and Viability

Human studies provide indirect support for a disease-activity gradient, with a higher plasma K/T ratio and generally greater IDO1 expression in active TB than in latent tuberculosis infection (LTBI), although differences in IDO1 expression were heterogeneous across study populations [19,37].

In mouse cellular models, IDO1 expression increased with higher Mtb multiplicity of infection and longer infection duration, providing direct experimental support for a burden- and time-dependent pattern of pathway activation [33]. Other animal models provide complementary support for burden-dependent expression of IDO1 in both macaques and mice; it was observed that non-pathogenic mycobacterial strains caused lower IDO1 expression [61].

The association between IDO1 expression and mycobacterial burden is particularly evident in macaque models, where uncontrolled Mtb replication appears to be a major factor influencing IDO1 expression. In macaque granuloma models, IDO1 expression increased in association with higher Mtb burden, and a positive correlation between these parameters was identified [61,65]. This relationship was further supported by lower IDO1 expression after infection with non-pathogenic Mtb strains, including MtbΔsigH and MtbΔdosR, and after anti-TB treatment. A positive correlation between IDO1 expression and the expression of *trpA, trpB, trpD, and trpY* genes (involved in de novo TRP synthesis) further links host TRP catabolism with bacterial TRP-biosynthesis responses, consistent with a TRP-starvation model driven by increased KP activity [61].

Mycobacterial strain virulence also appears to influence KP activity, with more virulent strains associated with stronger activation of TRP metabolism [57]. Clinical data are consistent with greater KP activation in more severe or drug-resistant disease contexts: plasma IDO-related activity, measured as the K/T ratio, was higher in newly diagnosed pulmonary multidrug-resistant TB than in drug-sensitive TB, healthy controls, and lung cancer patients [38].

Animal evidence further supports this relationship: in mice, infection with a more virulent Mtb strain produced greater and faster maximal IDO1 expression than infection with a standard strain [67]. Mycobacterial viability also appears to influence pathway activation, as live bacteria induced stronger IDO1 expression than heat-killed or irradiated bacteria [30].

#### 3.2.3. Additional Molecular Regulators

Beyond IFN-γ, other factors such as burden, virulence, or IFN-induced proteins with tetratricopeptide repeats (IFITs) signaling may also regulate IDO1 expression. In the human monocytic leukemia cell line (THP-1), plasmid-mediated overexpression of IFIT1/2/3 was associated with a significant increase in IDO1 expression, whereas IFIT knockdown produced the opposite effect, indicating that IFITs can regulate IDO1 expression in this in vitro model [56]. MET-dependent signaling appears to positively regulate IDO1-associated monocyte responses. Hepatocyte growth factor (HGF)—mediated MET activation increased IDO1 expression, whereas it was reduced by MET blockade or pharmacologic inhibition, supporting a model in which MET signaling may promote the IDO1-associated arm of the KP [60]. Interestingly, stimulation with the latency-associated antigen Rv1737c was related to an increased IDO1 expression in macrophage-like cells, suggesting that latent-infection-associated antigenic stimulation may also engage this pathway [55].

Enhanced IDO1 mRNA stability may represent one mechanism by which Mtb increases KP activity. In a model with inhibited transcription (via actinomycin D), IDO1 mRNA degradation was significantly lower in Mtb-infected cells than in uninfected cells, indicating that infection promotes IDO1 expression by increasing its mRNA stability [33]. Host genetic variation may also modulate KP activity in TB. IDO2 single-nucleotide polymorphisms (SNPs), particularly R248W and Y359X, have been associated with altered IDO1 expression and changes in the K/T ratio in patients with TB, suggesting that IDO2 genetic variation may influence IDO-related immunoregulatory signaling and downstream immune responses [28].

Taken together, the available evidence indicates that KP activation in TB is not controlled by a single upstream signal. IFN-γ has the strongest mechanistic support as a major inducer of IDO1, whereas mycobacterial burden, infection duration, viability and virulence appear to modulate the magnitude of this response, with additional contributions from host signaling pathways and genetic variation. However, the evidence for these additional regulators is derived largely from individual experimental models, and their relative importance in human TB remains uncertain. This complexity is important when interpreting differences in KP activity between patients or disease states, because variation in pathway readouts may reflect differences in both inflammatory signaling and pathogen–host context rather than a uniform response to Mtb infection.

### 3.3. Immunological Consequences of KP Activation

**Figure 3:**
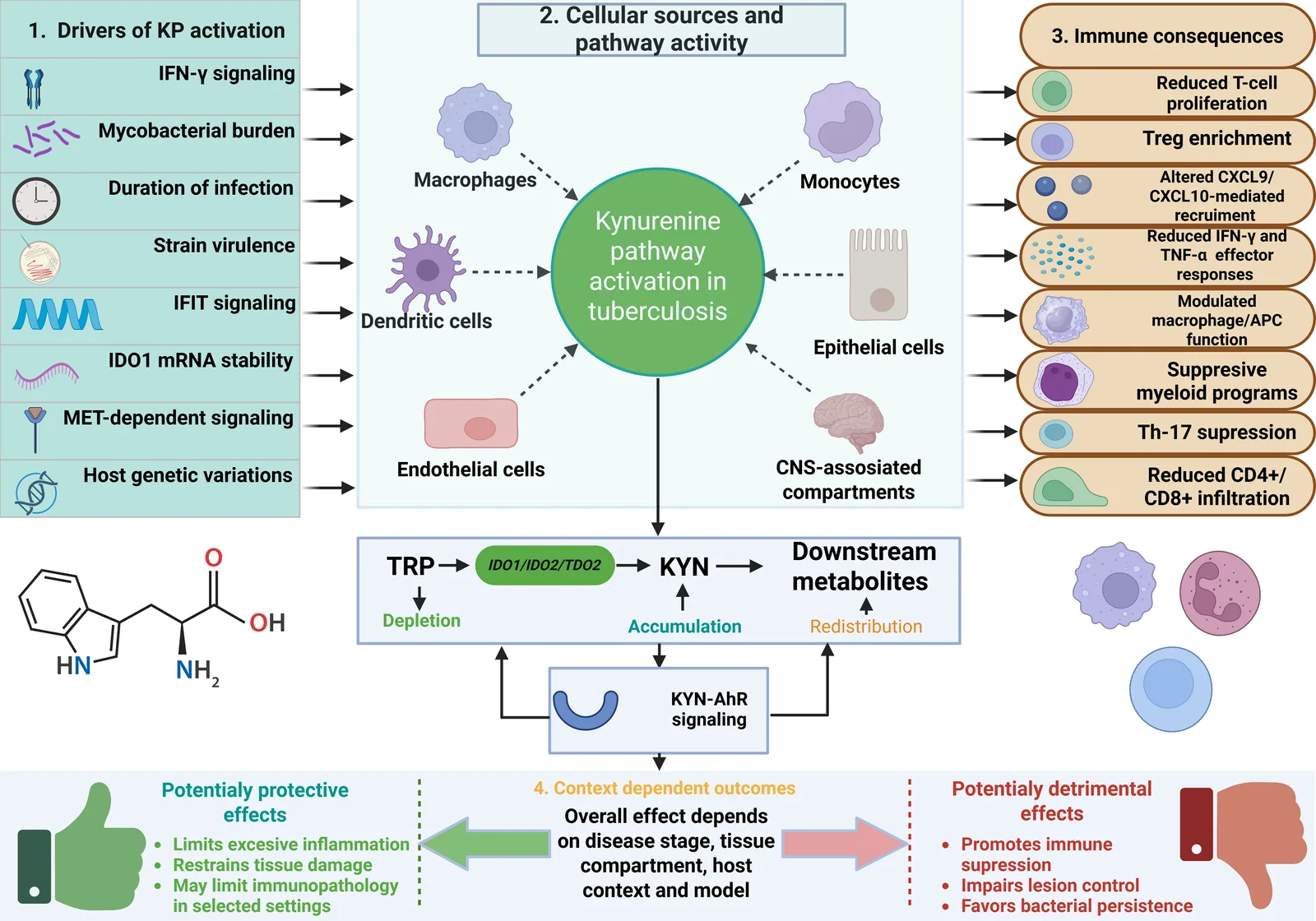
**Integrated model of KP activation and immune regulation in TB.** *M. tuberculosis* infection activates the KP through converging inflammatory, microbial, and host regulatory signals, including IFN-γ, mycobacterial burden, infection duration, strain virulence, and additional molecular regulators [14,19,54,56,57,60,61,65,67,68,72]. Pathway activation in myeloid, antigen-presenting, epithelial, endothelial, and CNS-associated compartments leads to TRP depletion, KYN accumulation, downstream metabolite redistribution, and KYN–AhR signaling [14,18,33,35,48,49,54,57,58,60,65,66,68,71,72]. These processes may suppress effector T-cell responses, alter chemokine-mediated recruitment, promote regulatory immune programs, and reshape macrophage and APC function [23,27,30,33,36,55,58,60,61,62,66,74,75,76,77,78,79]. The net effect is context-dependent and may contribute either to immune suppression and bacterial persistence or to limitation of excessive tissue-damaging inflammation [14,61,62,65,67,68]. Abbreviations: AhR, aryl hydrocarbon receptor; APC, antigen-presenting cell; CD4, cluster of differentiation 4; CD8, cluster of differentiation 8; CNS, central nervous system; CXCL9, C-X-C motif chemokine ligand 9; CXCL10, C-X-C motif chemokine ligand 10; IDO1, indoleamine 2,3-dioxygenase 1; IDO2, indoleamine 2,3-dioxygenase 2; IFIT, interferon-induced protein with tetratricopeptide repeats; IFN-γ, interferon gamma; KP, kynurenine pathway; KYN, kynurenine; MET, mesenchymal–epithelial transition factor receptor; mRNA, messenger RNA; TB, tuberculosis; TDO2, tryptophan 2,3-dioxygenase 2; Th17, T helper 17; TNF-α, tumor necrosis factor alpha; Treg, regulatory T cell; TRP, tryptophan. Created in BioRender. Stasiak, P. (2026). https://BioRender.com/3gb5wqi.

The host response to Mtb infection depends on a balance between protective antimicrobial immunity and mechanisms that limit inflammation-related tissue damage. KP activation appears to be involved in the regulation of immune equilibrium during TB. Available evidence links KP activity and its metabolites with changes in cellular immunity, APC function, and regulatory immune programs. Although these findings come from heterogeneous human, animal, and in vitro models, they collectively suggest that increased IDO-related activity is associated with a more immunoregulatory immune environment. This integrated model of KP activation, cellular sources, immune consequences, and context-dependent outcomes is summarized in Figure 3.

In non-TB settings, IDO-mediated TRP catabolism and KP metabolites have been shown to suppress proliferating T cells, induce apoptosis of activated T-cell subsets, and promote tolerogenic dendritic-cell and regulatory T cell (Treg)—associated immune programs [23,74,75,76,77,78]. The studies reviewed in this section therefore address how these established immunometabolic mechanisms operate specifically during Mtb infection.

### 3.4. Mechanistic Limitations of Methyltryptophan-Based IDO Inhibition

Several studies discussed below used methyltryptophan-based compounds as pharmacologic approaches to perturb IDO-related tryptophan catabolism, including D-1-methyltryptophan (D1-MT), racemic 1-methyl-DL-tryptophan (1-MT-DL), and compounds reported more generally as 1-MT. These compounds should not be regarded as uniformly selective direct inhibitors of IDO1 catalytic activity. D1-MT can counteract downstream consequences of tryptophan depletion by restoring mTOR signaling rather than acting solely through direct enzymatic inhibition of IDO [80]. In addition, 1-MT-DL has been shown to activate AhR-dependent signaling in mesenchymal stromal cells independently of IDO catalytic function [81]. Accordingly, biological effects observed after treatment with methyltryptophan-based compounds cannot be attributed unequivocally to selective inhibition of IDO1 catalytic activity and are therefore interpreted here as pharmacologic perturbation of IDO/KP-related immunoregulation.

### 3.5. Suppression of T-Cell Responses

In tuberculous granulomas, Treg enrichment correlated with the presence of IDO1^+^ cells, while IDO1 expression was inversely correlated with CD4^+^ and CD8^+^ T-cell infiltration in lung tissue from patients with high KYN levels [18,33]. Additionally, high KYN levels in peripheral blood showed a similar relationship with T-cell infiltration [33]. These data collectively suggest that heightened activity of the IDO1-KYN axis is linked to an enrichment of Treg cells and a reduction in CD4^+^ and CD8^+^ effector T-cell signatures, indicating a shift toward an immunosuppressive immune response. Similarly, C-X-C motif chemokine ligand 9 (CXCL9) and C-X-C motif chemokine ligand 10 (CXCL10) mRNA levels positively correlated with T-cell infiltration, suggesting that these chemokines may act as downstream mediators linking KP activity with local T-cell recruitment [33].

Human vaccine data further support an association between systemic IDO-related activity (measured as K/T ratio) and weaker antigen-specific T-cell responses in BCG-vaccinated individuals (modified vaccinia Ankara virus expressing antigen 85A—MVA85A). Higher baseline systemic K/T ratio was negatively associated with vaccine-induced antigen-specific IFN-γ responses, suggesting that pre-existing KP activity may limit CD4^+^ T-cell memory reaction [36]. Moreover, in TB-infected patients, elevated KYN levels in blood were linked to lower expression of effector cytokines, such as IFN-γ and tumor necrosis factor-α (TNF-α), by T cells and showed a positive correlation with the expression of inhibitory receptors (programmed cell death protein 1 (PD-1), T-cell immunoglobulin and mucin-domain-containing protein 3 (TIM-3), and *lymphocyte activation gene 3* (*LAG-3*) on T cells [33].

Human in vitro data provide more direct functional evidence that IDO-related activity can suppress T-cell proliferation. This suppressive effect appears to be mediated by KP activity and its downstream metabolites. In a separate human in vitro system using monocyte-derived APCs, pharmacologic IDO inhibition with 1-MT-DL increased proliferation of both CD3+CD4+ and CD3+CD8+ T cells responding to mycobacterial antigens, and addition of KP metabolites reversed the proliferative gain, directly linking IDO-related activity to suppression of both major T-cell lineages [30]. This is consistent with human vaccine data showing that higher KP activity is associated with weaker antigen-specific T-cell responses [36]. Together, these findings support a negative-feedback model in which IFN-γ induces IDO1 expression, whereas increased KP activity suppresses T-cell proliferation [30] and is associated with weaker antigen-specific IFN-γ responses [36].

In Mtb-infected mice treated with KYN, increased inhibitory receptor expression was accompanied by decreased CD4^+^ and CD8^+^ T-cell infiltration in the lungs and reduced T-cell release of IFN-γ and TNF-α [33]. However, another mouse model showed no difference in the number of activated CD4^+^ and CD8^+^ T cells in the lungs of wild-type and IDO1-deficient mice, indicating that the impact of IDO1 on pulmonary T-cell activation may depend on the model, timing, and experimental context [14].

Pharmacologic IDO inhibition with D1-MT in macaque TB models supports the idea that the IDO-KYN axis acts as a brake on protective cellular immunity. In these models, reducing IDO-related activity and IDO-driven TRP breakdown was linked to enhanced T-cell activation and proliferation programs, improved lesion control, and a more type 1 T helper cell (Th1)—supporting immune profile. Across macaque models, the most consistent functional signal was an increase in the proliferation marker Ki-67 (Ki-67) T cells, especially in BAL. However, total CD3^+^, CD4^+^, and CD8^+^ cell frequencies remained stable [61,62].

In macaque models, increased apoptosis and turnover among recruited T cells, including activation of apoptosis-related modules and nuclear receptor subfamily 4 group A member 1 (NR4A1) upregulation, may explain why enhanced proliferation coexisted with relatively stable overall T-cell numbers [61]. At the subset level, IDO inhibition was associated with a shift toward central- and effector-memory phenotypes and higher Ki-67 expression across memory compartments in the lung, BAL and blood. Total CD4^+^ and CD8^+^ counts remained mostly unchanged, apart from a modest endpoint increase in CD8^+^ effector-memory cells, suggesting qualitative remodeling of the T-cell pool rather than simple expansion [61].

In the setting of adjunctive chemotherapy with moxifloxacin, ethambutol, and D1-MT, endpoint flow-cytometric analysis showed few differences in lung T-cell subset percentages, whereas changes in BAL were more evident. These included increased BAL CD4^+^ effector T-cell percentages and proliferative signals in CD4^+^Ki-67^+^ and CD8^+^Ki-67^+^ cells in treated animals [62].

Transcriptomic data from macaque models further support functional remodeling of T-cell immunity after IDO inhibition. Reduced IDO1 expression was accompanied by lower expression of inhibitory or exhaustion-associated genes, including *LAG-3* and cytotoxic T-lymphocyte-associated protein 4 (CTLA-4), together with stronger T-cell stimulation, Th1-supporting modules such as C-C chemokine receptor type 5 (CCR5)/chemokine and nuclear factor of activated T cells (NFAT) pathways, and increased antimicrobial programs involving cathepsin, cytotoxic, and innate antimicrobial responses [61].

Overall, the evidence supports an immunoregulatory effect of the KP on T-cell responses in TB, but the strength of this conclusion differs across experimental settings. Human studies largely demonstrate associations between KP-related readouts and impaired T-cell responses, whereas more direct functional evidence comes from in vitro systems and pharmacologic animal models. Importantly, the absence of a clear T-cell phenotype in one IDO1-deficient mouse model, together with relatively stable total T-cell numbers despite functional and spatial changes in macaques, suggests that KP activity may influence T-cell quality, localization, and proliferative state more consistently than absolute T-cell abundance.

### 3.6. KYN–AhR Signaling in Cytokine and Chemokine Regulation

AhR is a ligand-activated transcription factor that translates environmental and endogenous metabolic signals into changes in gene expression. KYN and selected downstream KP metabolites, including KYNA and cinnabarinic acid, can activate AhR and influence Treg/ type 17 T helper cells (Th17) differentiation, IL-22 and inflammatory cytokine programs, and dendritic-cell immunogenicity [16,79,82,83,84]. These observations provide a broader mechanistic framework for examining how AhR-dependent signaling operates during Mtb infection.

Human cell-based TB models indicate that Mtb engages AhR-dependent signaling through context-dependent mechanisms. In one setting, IDO1-derived KYN activated AhR and suppressed signal transducer and activator of transcription 1 (STAT1)-dependent CXCL9/CXCL10 production, thereby limiting T-cell recruitment. In another study, AhR engagement in infected macrophages modified broader innate cytokine and inflammatory signaling programs [33,58].

In an IFN-γ-primed THP-1 macrophage model, Mtb infection activated the KYN–AhR axis, and AhR activity suppressed the STAT1–CXCL9/CXCL10 chemokine program involved in CD4^+^ and CD8^+^ T-cell recruitment. Mechanistically, AhR upregulated suppressor of cytokine signaling 3 (SOCS3) (including direct promoter engagement), which in turn dampened Janus kinase (JAK)—STAT1 phosphorylation, reducing CXCL9/CXCL10 expression. Functionally, AhR knockdown or pharmacologic inhibition increased STAT1 activation, restored CXCL9/CXCL10, and enhanced T-cell chemotaxis, while SOCS3 knockdown similarly increased CXCL9/CXCL10 and T-cell migration capacity [33]. These findings were recapitulated in vivo, where the KYN-AhR axis regulated CXCL9/CXCL10 levels and pulmonary T-cell recruitment without altering C-X-C motif chemokine receptor 3 (CXCR3) expression on CD4^+^ or CD8^+^ T cells [33].

A distinct AhR-linked output was observed in THP-1 cells and primary human macrophages infected with Mtb, where infection strongly induced IDO1, TDO2, and other KP enzymes together with robust AhR pathway engagement, including increased receptor/partner expression and deoxyribonucleic acid (DNA) binding activity. In this setting, AhR promoted transcriptional programs linked to pro-inflammatory cytokines, including the *interleukin-1 beta gene (IL1B)*, as supported by AhR/AhR nuclear translocator binding to regulatory elements and attenuation after AhR knockdown or antagonism. IL-23 was also induced during infection in parallel with IDO1/TDO2 expression; exogenous KYN amplified the *IL-23 subunit alpha gene (IL23A)*, which encodes the p19 subunit of IL-23, whereas AhR antagonism reduced IL23A expression. These data further support IL23A as a direct AhR target gene through near-upstream enhancer/AhR response element regions in human macrophage systems. Induction of IL-1β and IL-23 in macrophages may promote proliferation of IL-22-producing lymphoid cells and increase IL-22 secretion, thereby supporting epithelial host defense at barrier sites. Moreover, AhR-deficient THP-1 cells showed lower expression of IDO1, IDO2, and TDO, supporting a positive-feedback loop between AhR signaling and KP enzyme expression [58]. Similar coupling between AhR signaling and KP activation has also been observed in a TBM context [48].

These findings identify AhR as an important downstream mediator of KP-related immune regulation in TB. However, the outcome of AhR-mediated effects is divergent. Depending on the experimental context, AhR signaling can suppress chemokine programs involved in T-cell recruitment while promoting selected inflammatory cytokine responses, suggesting that its functional consequences depend on cell type, activation state, and the surrounding inflammatory milieu. Although pharmacologic and genetic interventions provide stronger mechanistic evidence than measurements of metabolites alone, most available data derive from macrophage-based experimental systems, and the relative importance of these AhR-dependent programs in human TB lesions remains to be established.

### 3.7. Effects on Monocytes, Macrophages, and APCs

Human monocyte and macrophage data indicate that IDO-related activity can restrain innate antimicrobial and APC functions. In human monocytes, IDO emerged as a negative regulator of antimicrobial activation. Upon mycobacterial stimulation, IDO1 expression was associated with a restrained innate response, whereas pharmacologic IDO inhibition with D1-MT led to a significant increase in mycobacterial uptake (GFP-BCG internalization) by CD14^+^ monocytes and concurrently enhanced expression of the costimulatory/activation markers CD40, CD83, and CD86 (increased mean fluorescence intensity) [60]. However, not all activation-associated findings are consistent, as increased IDO1 expression was also observed in activated macrophages with high CD40 and CD80 expression [55].

In murine APCs, IDO1 restrained mycobacteria-specific CD4^+^ T-cell proliferation in vitro. When bone marrow-derived DCs lacked IDO1, carboxyfluorescein succinimidyl ester (CFSE)—labeled P25 antigen 85-specific CD4^+^ T cells initiated division earlier and entered more rounds of proliferation than in wild-type bone marrow-derived dendritic cell (BMDC) cultures; pharmacologic inhibition with 1-MT-DL produced a similar enhancement [14]. A macaque macrophage–T-cell co-culture model provided additional functional support for this pattern: silencing IDO1 in macrophages improved control of Mtb replication in the presence of Mtb-specific CD4^+^ T cells [61].

These findings suggest that IDO-related activity can restrain selected antimicrobial and antigen-presenting functions of myeloid cells during mycobacterial infection. However, the available evidence is derived mainly from in vitro or ex vivo systems, and the observed effects are not uniform across activation states or experimental models. This indicates that IDO-related signaling is more likely to modulate the functional state of monocytes, macrophages, and APCs than to impose a single uniformly suppressive phenotype.

### 3.8. Regulatory and Immunosuppressive Immune Polarization

Experimental murine data suggest that KP activation can shape regulatory polarization by limiting Th17 responses and promoting Treg-associated immune programs. In vivo, the KP metabolites regulated Th17 cell development in an IL-23-dependent manner. L-KYN, 3′-OH-DL-kynurenine, 3-HAA, AA, and QUIN caused dose-dependent inhibition of IL-17 production by CD4+ T cells under Th-17 cell-differentiating conditions. This inhibitory effect was retained even when KP metabolites were added at later stages of Th17 differentiation [68].

A complementary murine dendritic-cell model showed that alpha-crystallin 1—conditioned DCs displayed increased IDO levels after IFN-γ stimulation together with higher expression of immunoregulatory cytokine transcripts, including IL-10 and transforming growth factor-β (TGF-β). In DC-T-cell cocultures, this was accompanied by expansion of CD4^+^CD25^+^Foxp3^+^ Tregs [71]. In vivo TB models are consistent with this regulatory pattern, linking KP activity with immunosuppressive cytokine signatures and reduced effector T-cell responses [33,67].

In the H37Rv mouse model, IDO inhibition with 1-MT-DL initiated during late infection reduced pulmonary bacillary burden at day 60, without significant changes in IFN-γ, TNF-α, or IL-4 expression or in the extent of pneumonia [67].

The immunoregulatory impact of IDO-related activity may also extend beyond T-cell polarization to the organization of granuloma-associated lymphoid structures. In macaque models, IDO inhibition was associated with more organized inducible bronchus-associated lymphoid tissue, larger and better-structured B-cell follicles, and increased intralesional B-cell presence, features often linked to protective immunity in TB lesions [61]. Taken together, these findings suggest that IDO-related activity can act as a negative regulator of host protection during Mtb infection by promoting T-cell dysfunction and less efficient lesion control. Conversely, blocking IDO shifts immunity toward more proliferative, better organized, and more antimicrobial lesion environments [61,62].

Combining findings from mouse and macaque models, the KP appears to influence the immune response toward an immunosuppressive or regulatory phenotype. Because non-human primate models more closely reproduce key features of human TB, these findings support the relevance of IDO-KYN-driven immune regulation for host–pathogen interactions in TB. However, the extent to which this translates into worse clinical outcomes in humans requires further validation [61,62].

### 3.9. Protective Versus Harmful Effects of KP Activation

Animal evidence on the role of KP activation in TB is heterogeneous and does not support a uniformly protective or uniformly detrimental interpretation. Available models include non-human primates, mainly rhesus and cynomolgus macaques, and several mouse strains, including C57BL/6, BALB/c, 129S2, and C3HeB/FeJ mice. A zebrafish larval model of *M. marinum* infection also showed reduced TRP levels, suggesting that altered TRP metabolism can occur across diverse mycobacterial infection models [31]. Interpretation is further complicated by differences in mycobacterial strain, host genetic background or genetic modification, infection stage, and experimental focus, since KP readouts were not always the primary endpoint. Macaque models are particularly relevant because they reproduce key pathological and immunological features of human TB more closely than most small-animal models [85]. Among mouse models, C3HeB/FeJ mice are particularly informative because they develop necrotic granuloma-like lesions that more closely resemble human TB pathology; however, this model was used only in a limited part of the available literature on this topic [61,86].

Mouse studies generally show that mycobacterial infection increases KP activity, as reflected by changes in pathway metabolites and increased expression or activity of enzymes such as IDO [14,28,30,31,67,68]. However, these models do not provide a consistent conclusion as to whether KP activation is ultimately beneficial or detrimental for host immunity during TB. The apparent role of KP activation in mouse TB models appears to depend strongly on the experimental context. In one mouse model, IDO1 deficiency did not alter immune control of Mtb or survival of infected animals. Pulmonary inflammation and survival were comparable between wild-type and IDO1-deficient mice [14]. Another mouse model indirectly suggested a protective role for IDO1 expression in non-hematopoietic cells during chronic TB, as animals with preserved or higher pulmonary IDO1 expression survived longer. However, interpretation of this finding is limited because reduced IDO1 expression resulted from IFN-γ unresponsiveness of non-hematopoietic lung cells, meaning that other IFN-γ-dependent mechanisms could also have contributed to the survival phenotype [68]. A more nuanced mouse model suggested that the consequences of KP activity may depend on disease severity and mycobacterial virulence. During infection with a less virulent Mtb strain, IDO-related activity enhanced bacterial growth in late PTB, supporting a detrimental role for pathway activation in this context. In contrast, in infections with the hypervirulent 5186 strain, disrupting regulatory pathways (IDO and heme oxygenase-1(HO-1)) was associated with severe lung pathology and/or worse bacterial control. Combined inhibition of IDO and HO-1 (by 1-MT-DL plus zinc protoporphyrin IX) increased pulmonary bacillary loads at day 28 and was accompanied by pneumonic lesions containing necrotic foci with reduced TGF-β expression. These findings suggest that, in hypervirulent TB, IDO-associated regulatory pathways may help limit excessive tissue damage. However, this interpretation remains limited because IDO inhibition alone was not assessed in the hypervirulent-strain setting [67].

Taken together, studies suggest that KP activation in TB cannot be classified as uniformly protective or detrimental. Instead, its biological effect appears to depend on host species and genetic background, mycobacterial virulence, disease stage, tissue pathology, and whether IDO-related activity primarily limits antimicrobial immunity or restrains excessive inflammation.

### 3.10. KP in Tuberculous Granulomas

#### 3.10.1. Granuloma Architecture and Spatial Distribution of IDO1 Expression

In C57BL/6 mice with experimental TB, IDO1 levels were significantly upregulated in pulmonary lesions [30]. Additional murine lung-tissue studies also support infection-associated increases in IDO1 levels or KP activation, although they were not designed to resolve granuloma-specific spatial distribution [33,67]. Granuloma-level spatial evidence is more clearly defined in macaque TB models, including both cynomolgus and rhesus macaques. Across these macaque models, IDO1 levels were upregulated in granuloma-associated cellular compartments, supporting a prominent role for the KP within the granulomatous microenvironment [61,65,66].

In macaque models, tuberculous granulomas can be conceptualized as organized lesions composed of a lymphocytic cuff, an inner myeloid ring, and a necrotic core [61,65,66]. The necrotic core is composed predominantly of neutrophils, with an almost complete absence of polymorphonuclear myeloid-derived suppressor cells (PMN-MDSCs), and this region did not show a significant increase in IDO1 levels [66]. In contrast, the inner myeloid layer consists mainly of myeloid cells and is characterized by strong IDO1 abundance, which co-localizes with CD68+/CD163+ macrophage signals [61,65,66]. The outer lymphocytic cuff is composed primarily of lymphocytes and PMN-MDSCs; notably, PMN-MDSCs located within this cuff exhibited higher IDO1 expression [61,66].

#### 3.10.2. IDO1, Programmed Death-Ligand 1(PD-L1), and Immunoregulatory Microenvironments in Human Granulomas

Human spatial profiling further extends this layered view by identifying stromal and tissue-associated microenvironments within tuberculous granulomas [18]. In this analysis, granulomas were segmented into distinct microenvironments, defined as cellular subsets co-occurring within spatial proximity, and the expression of the immunoregulatory molecules programmed death-ligand 1 (PD-L1) and IDO1 protein was quantified across specific myeloid cell subsets within each microenvironment [18]. These microenvironments included myeloid core-associated regions, lymphoid cuff and tertiary lymphoid structure-like regions, and stromal or tissue-associated niches such as fibrotic, vascular, and epithelial-parenchymal compartments [18]. For the immunoregulatory analysis relevant here, four microenvironments were particularly informative: MEMcore1, a macrophage- and monocyte-rich cellular neighborhood associated with the compact myeloid core of the granuloma; MELcuff, corresponding to the surrounding lymphocyte-rich cuff dominated by CD4^+^ and CD8^+^ T cells; MEIntMono, enriched in CD14^+^CD16^+^ intermediate monocytes; and MEFib, a fibrotic niche containing fibroblasts and CD163^+^ macrophages [18]. Importantly, the abundance of immunoregulatory markers differed substantially between these microenvironments. Among CD163^+^ macrophages, the percentage of PD-L1^+^ cells was 53.5% in MEMcore1, 71.6% in MELcuff, and 78.3% in MEIntMono, despite CD163^+^ macrophages themselves being most prevalent in MEFib. Similarly, IDO1^+^ CD11c^+^ dendritic cells were particularly enriched in MEFib (78.9%) and MEIntMono (83.7%) [18]. A key implication of this study is that microenvironmental location, rather than cellular abundance alone, shapes whether myeloid cells acquire an immunoregulatory phenotype. Taken together, the spatial co-expression of PD-L1 and IDO1 defines a coordinated immunoregulatory feature within TB granulomas, consistent with the idea that localized, myeloid-driven immune suppression can be concentrated in specific granuloma regions. When lymphocyte distribution was examined across microenvironments, Treg cells were enriched in MEMcore1 relative to the lymphocytic cuff, and the number of MEMcore1-infiltrating Treg cells correlated with both IDO1^+^ and PD-L1^+^ cells [18].

#### 3.10.3. Granuloma IDO1 Expression, Bacterial Burden, and Disease Stage

Within granulomatous lung tissue, IDO1 expression appears to vary with disease stage, infection status, mycobacterial burden, and vaccination history. Macaque models consistently show increased pulmonary or granuloma-associated IDO1 expression after Mtb infection [61,65,66]. In macaque models comparing disease states, IDO1 expression was higher in active TB than in latent infection [61,66]. Consistent with this pattern, simian immunodeficiency virus (SIV) co-infection associated with TB reactivation was accompanied by elevated IDO1 expression levels [61]. Vaccination and infection stage also influenced IDO1 expression in macaque lung lesions [65]. Compared with normal lung tissue, IDO1 gene expression in early granulomas at 5 weeks post-infection was approximately 122-fold higher in sham-vaccinated macaques and 22-fold higher in BCG-vaccinated animals. At 10 weeks post-infection, IDO1 expression remained elevated, reaching approximately 26-fold above normal lung levels in sham-vaccinated animals and 8-fold in BCG-vaccinated animals [65]. Within granulomatous lesions, IDO1 expression also appears to track with local Mtb burden, linking KP activation to the intensity of bacillary replication in the lesion microenvironment [61,65]. In addition, IDO1 expression positively correlated with bacterial trpA, trpB, trpD, and trpY expression. This association is consistent with a TRP-starvation model in which increased host KP activity imposes TRP-limiting pressure that is accompanied by compensatory activation of de novo TRP biosynthesis in Mtb [61].

#### 3.10.4. Functional Consequences of IDO Inhibition Within Granulomas

**Figure 4:**
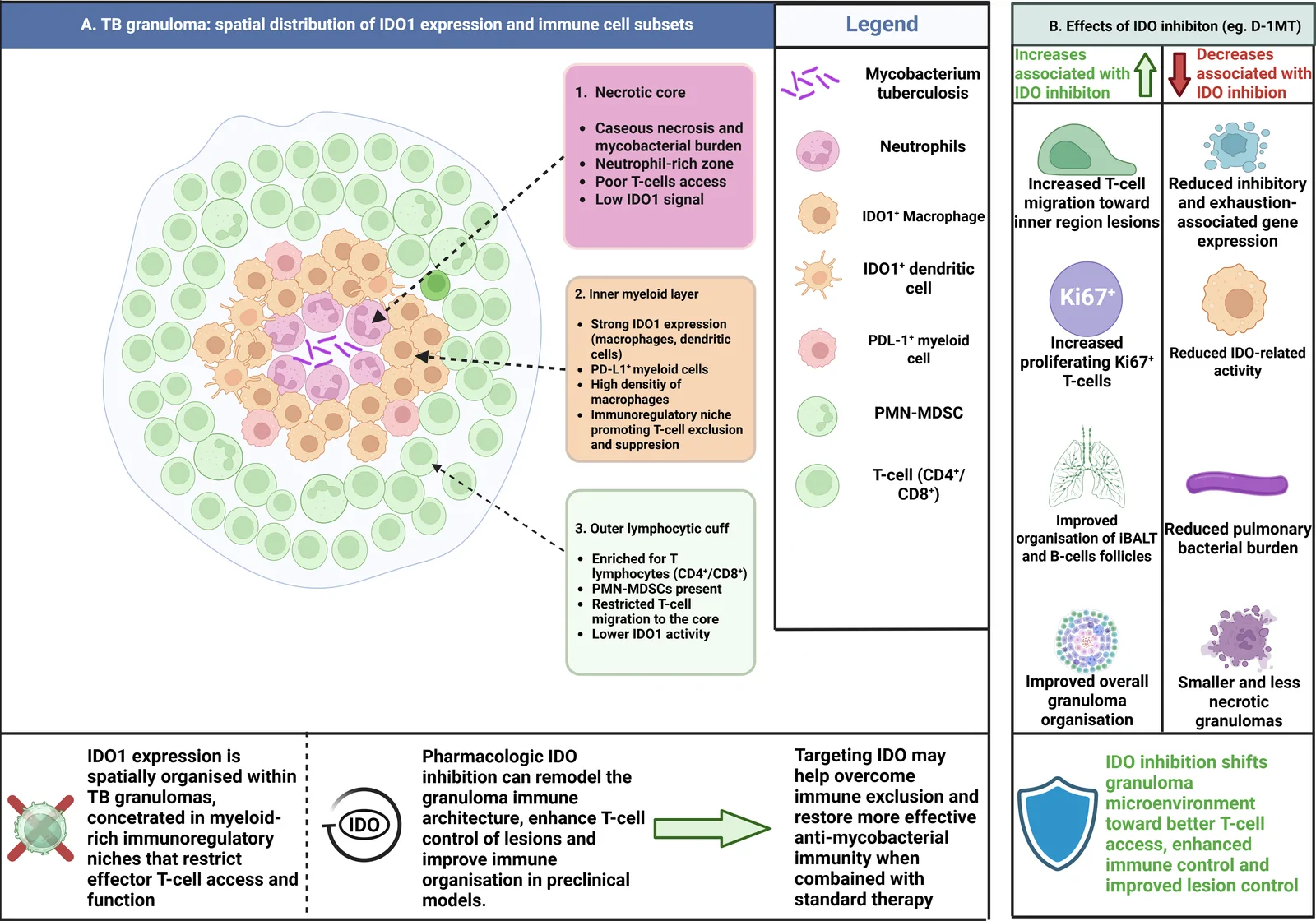
**Spatial organization of IDO-related immunoregulation in tuberculous granulomas.** This scheme integrates evidence from macaque granuloma models, human spatial profiling of tuberculous granulomas, and preclinical IDO-inhibition studies. It is intended as a conceptual model rather than a quantitative representation of a single experimental system. (**A**). Tuberculous granulomas can be conceptualized as spatially organized lesions containing a necrotic core, an inner myeloid layer, and an outer lymphocytic cuff [18,65]. IDO expression is enriched in myeloid-rich regions, particularly macrophage-associated compartments, and overlaps with immunoregulatory features [18,65]. This spatial organization may limit effective T-cell access to lesion centers. (**B**). Pharmacologic IDO inhibition in macaque models increases T-cell movement toward necrotic regions and is associated with qualitative remodeling of granuloma-associated immunity [61,62]. Abbreviations: CD4, cluster of differentiation 4; CD8, cluster of differentiation 8; D-1MT, D-1-methyltryptophan; iBALT, inducible bronchus-associated lymphoid tissue; IDO, indoleamine 2,3-dioxygenase; IDO1, indoleamine 2,3-dioxygenase 1; Ki67, proliferation marker Ki-67; PD-L1, programmed death-ligand 1; PMN-MDSC, polymorphonuclear myeloid-derived suppressor cell; TB, tuberculosis. Created in BioRender. Stasiak, P. (2026) https://BioRender.com/idmtpze.

The functional relevance of granuloma-associated IDO-related activity was tested in macaque TB models using D1-MT, a pharmacologic IDO inhibitor. D1-MT treatment resulted in an increased number of CD3+ T cells per lesion and enhanced T-cell migration from the lymphocytic cuff toward the necrotic center of the granuloma. Importantly, quantification of IDO1^+^ cells showed that the number of IDO1+ cells in the macrophage layer was essentially unchanged between groups, suggesting that D1-MT did not reduce IDO1 levels per se, but rather diminished IDO-related activity. Collectively, these findings indicate that IDO-related signaling modulates T-cell infiltration into the necrotic centers of granulomas [61]. The spatial organization of IDO1 presence within tuberculous granulomas and the proposed effects of pharmacologic IDO inhibition are summarized in Figure 4. Taken together, granuloma studies indicate that IDO1-associated immunoregulation is spatially organized rather than uniformly distributed across tuberculous lesions. Human spatial profiling provides direct evidence of IDO1- and PD-L1-enriched myeloid microenvironments, whereas macaque studies add associations with disease stage and bacterial burden as well as intervention-based evidence that perturbing IDO-related activity can alter T-cell access to granuloma centers. However, the human evidence is largely cross-sectional and based on protein localization rather than direct measurement of local KP metabolic flux, while D1-MT is not a selective inhibitor of IDO1 catalytic activity; therefore, the available data support a spatially structured regulatory role for IDO-related signaling but do not establish that IDO1 activity alone determines granuloma outcome.

### 3.11. KP Across Specific TB Compartments and Clinical Contexts

#### 3.11.1. Tuberculous Pleural Effusion

Tuberculous pleural effusion represents a localized inflammatory compartment in which KP activation can be assessed directly. This condition can accompany both primary infection and disease reactivation; in adults living in high-income settings, reactivation is more commonly implicated. The effusion is generally attributed to rupture of a small subpleural tuberculous lesion with leakage of bacillary material into the pleural cavity [87].

Across studies assessing this compartment, tuberculous pleural fluid showed a pattern consistent with local TRP catabolism. Compared with peripheral blood, tuberculous pleural effusion showed higher KYN levels, lower TRP levels, and a higher K/T ratio [34]. Soluble IDO concentrations were also higher in pleural fluid than in peripheral blood. By contrast, circulating soluble IDO levels did not differ significantly between PTB and pleural TB, suggesting that the IDO signal in pleural disease may be compartmentalized rather than simply systemic [29].

Comparisons with non-tuberculous pleural effusions further support TB-specific activation of TRP catabolism in the pleural compartment. Tuberculous pleural effusions showed higher KYN levels and lower TRP levels than non-tuberculous pleural effusions [29,45]. A higher K/T ratio in tuberculous pleural effusion further supported increased local IDO-related TRP breakdown [46].

Functional data further indicate that IDO-related activity has immunosuppressive relevance in this compartment, as pharmacologic IDO inhibition with 1-MT restored IFN-γ production, suggesting that local IDO-related activity can suppress Th1-type immune responses in pleural fluid [46,47]. In addition, soluble IDO positively correlated with lactate dehydrogenase (LDH) in tuberculous pleural effusion, linking soluble IDO levels with local inflammatory or tissue-damage markers [29].

Pleural TB provides one of the clearest examples of compartmentalized KP-related alterations in human disease, because metabolic, protein, and functional readouts converge within the same anatomical compartment. However, the available studies do not resolve the cellular source or specific enzyme responsible for altered TRP catabolism, and neither soluble IDO concentrations nor the K/T ratio can be equated with local IDO1 catalytic activity. Thus, pleural fluid represents a valuable compartment for studying local KP biology, but findings obtained in this setting should not be extrapolated directly to systemic circulation or pulmonary parenchymal disease.

#### 3.11.2. Tuberculous Meningitis (TBM) and Other TB-Induced Central Manifestations

TBM represents a severe CNS manifestation of TB in which KP activity appears to be strongly altered. Available evidence consistently suggests that this pathway contributes to the inflammatory and metabolic environment of TBM. In cerebrospinal fluid (CSF), TRP concentrations were decreased, whereas KYN and other downstream KP metabolites were increased [35,41,48,49]. Within the CNS, TRP catabolism through the KP can involve both IDO1 and IDO2. Differences in the regulation and tissue distribution of these two isoenzymes are therefore important for understanding compartment-specific KP activity in TBM [49].

Human CNS tissue analyses further indicate that KP activity in TBM is not restricted to CSF, but can also be detected in the parenchyma, choroid plexus, inferior olive, and cerebellum, particularly in Purkinje cells and the granular layer [48].

##### CSF Changes in KP Metabolites 

In TBM, CSF TRP levels were negatively correlated with IFN-γ, consistent with cytokine-driven activation of IDO-related TRP catabolism. Several KP metabolites, including KYN, KYNA, 3-HAA, and QUIN, were positively associated with a broad panel of inflammatory proteins, particularly TNF-α [35].

Beyond cytokine-driven induction of KP enzymes, inter-individual differences in CSF TRP in TBM also appear to reflect host genetic variation. Genome-wide SNP-based mapping of TRP quantitative trait loci identified 11 independent loci with suggestive associations with CSF TRP concentrations, although no single variant reached genome-wide significance. These findings suggest that baseline TRP availability within the CSF may be partly influenced by host genetics, potentially contributing to the wide variability in TRP depletion observed among patients with TBM [49].

##### IDO1 and IDO2 Distribution in CNS TB

IDO1 protein was detected in granulomas and leptomeningeal regions, with a predominantly perivascular pattern between the brainstem and cerebellum. In non-necrotizing granulomas, IDO1 expression was intense and localized, while in necrotizing granulomas, it appeared more spread out. IDO1 expression was also detected outside granulomas, including in the brain parenchyma and inferior olivary nuclei, indicating that IDO1 expression may extend beyond granulomatous lesions [48].

IDO2 expression partly paralleled the granulomatous IDO1 expression. However, IDO2 was also highly expressed in brain regions without granulomas, including the parenchyma, choroid plexus, inferior olive, and cerebellum, particularly in Purkinje cells and the granular layer. Additionally, QUIN and 3-HAA, key products of the kynurenic pathway, were strongly present in the cytoplasm of cells expressing IDO1 and IDO2, as well as in cells not expressing these enzymes. These metabolites were observed within granulomas, in nearby blood vessels, and in regions without granulomatous inflammation. Together, these findings suggest that KP activation and metabolite accumulation in TBM are spatially widespread and may affect both granulomatous and non-granulomatous CNS regions [48].

Regulation of IDO2 appears to differ from that of IDO1: whereas IDO1 is classically linked to IFN-γ- and inflammatory cytokine-driven induction, IDO2 regulation may involve AhR-dependent mechanisms [88,89]. This creates the potential for a positive-feedback loop between KP metabolite production and AhR signaling, which may be limited by the AhR repressor [48,90,91]. This regulatory distinction may help explain the different spatial patterns of IDO1 and IDO2 in CNS TB: IDO1 expression appears more closely linked to inflammatory foci, whereas IDO2 expression may be influenced by diffusion of AhR ligands and local modulation of AhR-repressor activity. Additionally, AhR was found in the nuclei of IDO2-expressing cells, indicating its activation and suggesting a role in promoting IDO2 expression [48].

##### Blood–Brain Barrier (BBB) Dysfunction and Peripheral Contribution to CSF KYN

The altered CSF K/T pattern observed in TBM is unlikely to result solely from intrathecal IDO1/IDO2 upregulation. The increase in CSF KYN was greater than the simultaneous decline in CSF TRP, indicating that elevated CSF KYN cannot be explained only by increased intrathecal TRP breakdown. One plausible explanation is BBB dysfunction: the association between CSF KYN and CSF total protein, a commonly used indicator of barrier leakage, supports the possibility that BBB disruption allows peripheral KYN to enter the CSF [35]. Together, these findings support a dual-mechanism model in which elevated CSF KYN in TBM may reflect both cytokine-driven activation of the KP within the CNS and increased blood-to-CSF transfer caused by BBB impairment [35]. By contrast, CSF TRP concentrations were not correlated with CSF total protein, suggesting that TRP depletion in TBM is less likely to be driven primarily by BBB leakage [35,49].

##### Neurotoxicity

The spatial distribution of IDO2 and downstream KP metabolites in TBM also suggests a possible link with apoptosis, autophagy, and neurotoxicity [48]. Potential mechanisms include inhibition of amino acid uptake by KP metabolites and accumulation of downstream products with neurotoxic potential, particularly QUIN [48]. Apoptosis and autophagy showed distinct spatial patterns: within granulomas in the leptomeninges and subarachnoid space, apoptotic cells were more frequent than autophagic cells. In contrast, autophagy rather than apoptosis was more prominent in the parenchyma. Notably, both autophagy and apoptosis were evident in the inferior olive, although autophagy was more pronounced [48]. Together, these findings suggest that KP activity in TBM may contribute not only to inflammatory immune regulation but also to compartment-specific CNS tissue injury.

##### Mood Disorders in TB

Beyond TBM, KP activation may also be relevant to neuropsychiatric manifestations associated with TB, particularly depressive symptoms. In a cohort of patients with TB, 73.5% had a Patient Health Questionnaire-9 (PHQ-9) score (a widely used, self-administered clinical tool for screening, diagnosing, and monitoring the severity of depression) above 5 [41]. Patients with depressive syndromes showed a biochemical profile consistent with increased IDO-related activity, including lower serum TRP levels, higher serum KYN levels, and an increased K/T ratio. The relationship between KP activity and depressive symptoms was further supported by correlations with PHQ-9 scores: serum KYN levels were positively correlated with PHQ-9 scores, serum TRP levels were negatively correlated with PHQ-9 scores, and the K/T ratio was significantly associated with PHQ-9 scores. These findings suggest that IDO-related TRP catabolism may contribute to depressive symptoms in TB, although causality cannot be established from these observational data [41].

##### Schizophrenia and TB

Some epidemiological and pharmacological observations suggest potential links between schizophrenia, antipsychotic treatment, KP metabolism, and TB. In a 12-year nationwide retrospective cohort study, patients with schizophrenia had a significantly higher adjusted incidence of newly diagnosed TB than the general population (HR = 1.52, 95% CI 1.29–1.79) [92]. However, this epidemiological association does not establish a shared biological mechanism between schizophrenia and TB. Antipsychotic treatment may also influence KP-related readouts. In a prospective study of patients with first-episode psychosis, seven months of antipsychotic treatment was associated with an increase in serum KYN from 2.20 to 2.86 μmol/L (*p* = 0.0003) [93]. Because several different antipsychotic agents were used in this naturalistic treatment setting, the observed metabolic change cannot be attributed to a specific drug.

The phenothiazine antipsychotic thioridazine has demonstrated direct antimycobacterial activity in experimental systems. In human monocyte-derived macrophages, thioridazine enhanced intracellular killing of both drug-susceptible and multidrug-resistant Mtb at concentrations considered clinically relevant by the investigators [94]. However, there is currently no direct evidence that the antimycobacterial activity of thioridazine is mediated through modulation of the KP. Therefore, the increased susceptibility to TB observed in schizophrenia, antipsychotic-associated changes in KYN levels, and the antimycobacterial activity of thioridazine should be regarded as potentially relevant but mechanistically distinct observations rather than evidence of a common KP-dependent mechanism.

Overall, the evidence for KP dysregulation in CNS-related TB is supported by complementary CSF metabolomic and tissue-localization studies, but its functional interpretation remains complex. In TBM, altered CSF metabolite concentrations may reflect both local pathway activity and peripheral contributions associated with blood–brain barrier dysfunction, while tissue expression of IDO1, IDO2, and downstream metabolites does not by itself establish their contribution to neurological injury. Evidence linking KP alterations to neuropsychiatric manifestations of TB is even more limited. Thus, the KP represents a plausible link between inflammation, metabolic remodeling, and CNS pathology in TB, but the relative contributions of local metabolism, systemic inflammation, barrier dysfunction, and direct neuroactive effects remain unresolved.

#### 3.11.3. HIV–TB Co-Infection

Clinical studies consistently link HIV infection with increased KP activity, most commonly reflected by a higher plasma K/T ratio as a proxy of IDO-related TRP catabolism [32,37]. Among people living with HIV (PLWH) who were naive to antiretroviral therapy (ART), with or without TB, the plasma K/T ratio positively correlated with HIV ribonucleic acid (RNA) and negatively correlated with CD4 counts, indicating that uncontrolled HIV replication and more advanced immunodeficiency are associated with increased TRP breakdown [32]. However, this relationship was not observed in another cohort, suggesting that TB-related immune activation may contribute to IDO-related activity independently of standard HIV disease markers [39]. In ART-treated participants, K/T ratios at TB diagnosis showed a non-linear relationship with CD4 counts, while individuals with treatment failure showed increased K/T ratios after TB treatment alongside declining CD4 counts. These findings link altered HIV immune control with KP activity in some patients [40]. A positive association with CXCL10 further links KP activity with chemokine-driven inflammatory signaling, consistent with the CXCL10-related immune programs discussed above [32,33].

In a diagnostic accuracy study including both HIV-uninfected and HIV-infected participants, HIV infection was independently associated with a higher K/T ratio in multivariable analysis, indicating that HIV status contributes to elevated KP activity beyond TB status and other covariates. Stratified analysis in this study also showed higher K/T ratios in active TB compared to latent infection within both HIV-negative and HIV-positive groups. The combined presence of HIV infection and TB was associated with an additive, or possibly synergistic, elevation in KP activity rather than a mutually exclusive effect. Accordingly, K/T ratios in both active TB and latent infection were higher in HIV-infected individuals than in HIV-uninfected individuals [37].

Within a cohort of HIV-infected adults, plasma IDO-related activity, estimated by the K/T ratio, increased in association with incident or active TB and was detectable months before TB symptoms appeared, suggesting potential predictive value [39]. In contrast, another ART-treated cohort did not show significant differences in K/T ratios during the six months before TB diagnosis, although higher ratios were observed at diagnosis compared with matched ART-treated controls [40].

Immunophenotyping in ART-treated PLWH and LTBI further supports a connection between increased TRP breakdown and HIV-related immune remodeling. In this setting, K/T ratios were higher in PLWH than in HIV-negative individuals and were inversely related to circulating Th17-related markers, linking HIV-associated KP activity with reduced Th17-associated immune signatures [42].

Transcriptomic analyses combining datasets with and without HIV co-infection indicate that IDO-pathway gene induction remains a feature of active TB regardless of HIV status, with IDO1 transcripts elevated in active TB in both HIV-negative and positive groups. IDO2 transcripts were increased mainly in HIV-positive individuals with active TB or latent infection, although at levels lower than IDO1, suggesting HIV co-infection influences which IDO family members are most induced [19].

Human macrophage models provide mechanistic support for this interpretation. In primary human monocyte-derived macrophages, HIV-1 infection alone caused only minor transcriptional changes but produced a detectable increase in IDO1 mRNA. By contrast, Mtb infection induced marked IDO1 upregulation, which was not significantly altered by HIV co-infection, suggesting that HIV may modestly prime the pathway whereas strong macrophage IDO1 induction during acute co-infection is largely driven by mycobacterial stimuli [59].

Pediatric household-contact data are consistent with this pattern: plasma IDO levels were highest among TB cases and heavily exposed household contacts, with a trend toward higher IDO levels in HIV-infected household contacts than in HIV-exposed uninfected contacts. This suggests that HIV may amplify IDO-related signaling in the setting of intense mycobacterial exposure [51].

A rhesus macaque model of Mtb/SIV co-infection provided intervention-based support for the relevance of IDO-related activity in this setting by testing D1-MT added to concurrent combination antiretroviral therapy (cART). D1-MT + cART reduced KYN levels in BAL cells relative to pretreatment, and quantitative BAL TRP and KYN measurements showed a further reduction in the K/T ratio, consistent with decreased IDO-related activity. In lung granulomas, confocal imaging and quantitative tissue analysis showed significantly reduced IDO1 levels in the D1-MT + cART group, and multiplex immunohistochemistry/quantification demonstrated fewer total IDO1-expressing cells and fewer IDO1-expressing macrophages in granulomas with D1-MT + cART compared with cART alone. Within granulomas, D1-MT plus cART was also associated with higher CD8^+^ T-cell frequencies, while CD4^+^ T-cell frequencies remained similar, linking reduced IDO-related activity or IDO1 expression with altered T-cell representation at the lesion site in this model [63].

HIV–TB co-infection illustrates both the biological relevance and the interpretative difficulty of KP-related readouts. HIV infection can independently increase TRP catabolism, making it difficult to separate HIV-driven immune activation from TB-specific pathway changes in peripheral blood, and associations with viral load or CD4^+^ T-cell counts are not fully consistent across cohorts. The macaque intervention data provide stronger evidence that modulation of IDO-related activity can alter local immune responses during Mtb/SIV co-infection, but these findings remain preclinical. Thus, KP-related biomarkers in HIV–TB should be interpreted in the context of HIV control, ART status, and disease compartment rather than as TB-specific indicators in isolation.

#### 3.11.4. Pediatric TB

Evidence for KP perturbation is also present in pediatric PTB cohorts assessed using blood metabolomics. In a targeted liquid chromatography–tandem mass spectrometry (LC–MS/MS) study of Polish children (2–15 years), pediatric patients with active TB and LTBI showed increased KYN levels in *Mtb* antigen-stimulated whole-blood cultures compared with healthy controls, whereas children with non-mycobacterial pneumonia showed reduced KYN in the same assay context. A similar pattern was observed for the K/T ratio [52]. In an independent pediatric cohort, targeted quantification controlling for age and study site showed significantly lower plasma TRP concentrations in children with confirmed TB than in those with unlikely TB [53].

Overall, pediatric data support altered TRP metabolism in TB, but the evidence remains limited and methodologically heterogeneous. The available studies assessed different biological matrices and experimental conditions, including Mtb antigen-stimulated whole blood and unstimulated plasma, and therefore their findings should not be interpreted as directly equivalent measures of systemic KP activity. Moreover, current pediatric evidence is primarily biomarker-based and does not establish the cellular sources or functional consequences of KP alterations. Further age-stratified studies integrating circulating metabolites with cellular and tissue-level readouts will be needed to determine whether pediatric KP signatures reflect disease-specific biology or broader developmental and inflammatory variation.

#### 3.11.5. TB and Lymphatic Tissue

Human lymph-node tissue provides additional evidence that KP-related immunoregulation extends beyond pulmonary and CNS compartments. In cervical lymph-node biopsies from patients with TB, with or without HIV co-infection, IDO1 mRNA and protein expression were significantly increased compared with control lymph nodes. IDO1 protein was detected predominantly within granulomatous regions and co-localized mainly with CD68^+^ macrophages, although some IDO1^+^CD68^−^ cells were also present. TB/HIV co-infection was additionally associated with poorly organized, necrotic granulomas, increased *Mtb* antigen burden, reduced lymphocyte representation, and enhanced immunosuppressive myeloid features. However, because TRP, KYN, and downstream KP metabolites were not measured, these findings demonstrate compartment-specific IDO1 mRNA and protein expression rather than complete KP metabolic activity [50].

Taken together, currently available evidence supports compartment-specific KP-related alterations in pulmonary and granulomatous tissue, pleural fluid, the CNS and CSF, and lymph nodes, with additional systemic evidence in HIV–TB co-infection and pediatric TB. No further organ-specific TB compartments with direct measurements of KP metabolites, enzymes, or related activity were identified in the literature reviewed.

#### 3.11.6. Potential Confounders in KP Biomarker Interpretation

**Figure 5:**
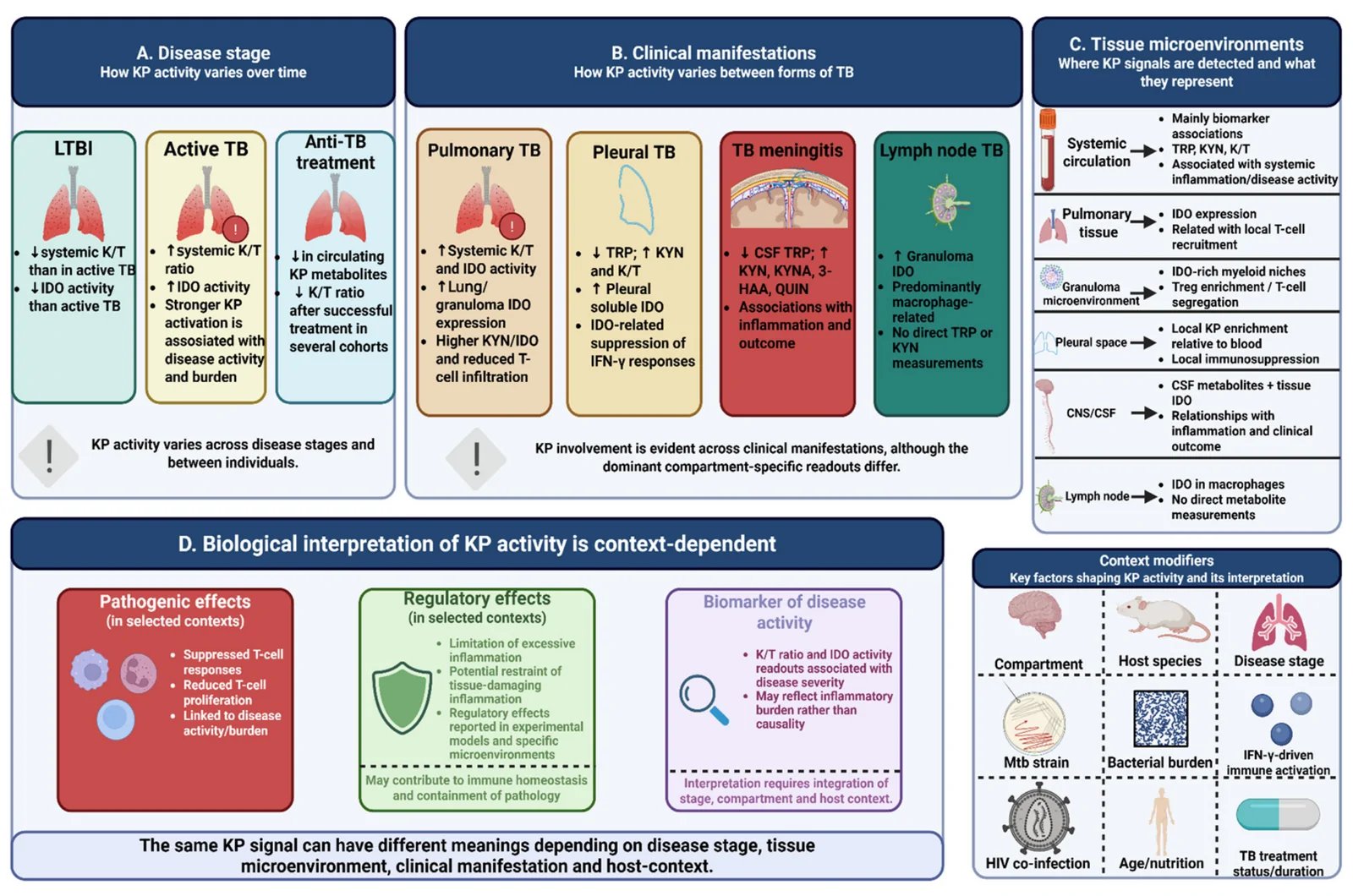
**Context-dependent kynurenine pathway activity across tuberculosis disease stages, clinical manifestations, and tissue microenvironments.** KP-related readouts differ between latent and active TB and may change during anti-TB treatment [19,31,37,39,40,41,43,61,65,66]. Distinct compartment-specific patterns have been reported in pulmonary and granulomatous tissue [18,33,61,65,66], pleural TB [29,34,45,46,47], CNS/CSF [35,48,49], and lymph-node TB [50]. Depending on the biological context, KP activity may be linked to immune-restrictive effects, regulatory control of inflammation, or may primarily reflect disease activity rather than directly drive pathology [14,33,61,62,67,68]. Its interpretation is further influenced by host species or experimental model, mycobacterial strain and burden, HIV co-infection, age, nutritional status, and treatment [19,32,43,53,57,61,65]. For graphical simplicity, IDO is used as an umbrella term encompassing study-specific measurements of IDO, IDO1 or IDO2 expression, soluble IDO, and IDO-related activity. Indirect readouts such as the K/T ratio do not distinguish the relative contributions of IDO1, IDO2, and TDO2. **Abbreviations:** 3-HAA, 3-hydroxyanthranilic acid; CNS, central nervous system; CSF, cerebrospinal fluid; HIV, human immunodeficiency virus; IDO, indoleamine 2,3-dioxygenase; IDO1, indoleamine 2,3-dioxygenase 1; IDO2, indoleamine 2,3-dioxygenase 2; IFN-γ, interferon gamma; KP, kynurenine pathway; K/T ratio, kynurenine-to-tryptophan ratio; KYN, kynurenine; KYNA, kynurenic acid; LTBI, latent tuberculosis infection; Mtb, *Mycobacterium tuberculosis*; QUIN, quinolinic acid; TB, tuberculosis; TDO2, tryptophan 2,3-dioxygenase 2; TRP, tryptophan. Created in BioRender. Stasiak, P. (2026) https://BioRender.com/5quehzv.

The interpretation of KP-related biomarkers in human TB should also account for host and clinical factors that may affect their specificity. HIV status represents an important potential confounder, as HIV infection has been independently associated with higher K/T ratios, and IDO-related activity has also been linked to HIV viral load and CD4^+^ T-cell counts [32,37]. Nutritional status may contribute additional variability, as inverse associations between the K/T ratio and nutritional indicators such as body mass index and mid-upper arm circumference have been reported in HIV-associated TB cohorts [32]. Age should also be considered when comparing biomarker profiles across populations, particularly in pediatric studies, where age-related metabolic variability may influence biomarker performance [53]. Accordingly, future biomarker-validation studies should prospectively record and, where appropriate, adjust for relevant host and clinical factors, including HIV status, nutritional status, age, and other major inflammatory or metabolic comorbidities. Importantly, associations between increased KP activity and TB severity should not be interpreted as evidence of causality. In human biomarker studies, changes in the K/T ratio, TRP and KYN concentrations, or IDO-related activity may have diagnostic or prognostic value while primarily reflecting the net effect of underlying inflammatory, immunological, and metabolic processes rather than representing a mechanistic driver of TB pathophysiology. Taken together, the available evidence indicates that the magnitude and biological interpretation of KP activity in TB depend on disease stage, clinical manifestation, tissue compartment, and host and pathogen context, as summarized in Figure 5.

#### 3.11.7. Association Versus Causation in KP Biomarker Studies

Most human evidence linking the KP to TB severity is observational. Consequently, elevated K/T ratios, reduced TRP concentrations, increased KYN concentrations, or increased IDO1 expression should primarily be interpreted as markers of a biological state rather than as direct evidence that the pathway causes disease progression. Active or severe TB increases bacillary burden, IFN-γ signaling, systemic inflammation, and tissue injury, all of which can induce TRP catabolism. Accordingly, reverse causation is plausible: greater KP activity may arise as a consequence of more intense disease rather than initiate or sustain that disease [19,37,38,61,65]. A decline in KP-related biomarkers during treatment may likewise reflect resolution of inflammatory signaling and reduction of bacterial burden rather than demonstrate that normalization of the KP mediates recovery.

The K/T ratio presents additional interpretative limitations. It is calculated from two circulating metabolite concentrations and does not directly measure intracellular pathway flux or distinguish the relative contributions of IDO1, IDO2, and TDO2 [13]. An elevated ratio may result from lower TRP, higher KYN, or both, and may be influenced by nutritional status, hepatic metabolism, renal function, HIV-related immune activation, age, and other inflammatory or metabolic comorbidities [13,32,37,53]. Compartment-specific measurements require similar caution. For example, CSF KYN in TBM may reflect both local synthesis and increased transport across a disrupted blood–brain barrier [35,49], whereas pleural-fluid changes cannot automatically be extrapolated to systemic or pulmonary-parenchymal KP activity.

Stronger causal inference would require evidence that KP activation precedes deterioration, that selective genetic or pharmacologic perturbation changes bacterial control or tissue injury, and that restoration of the relevant metabolite or signaling branch reverses the effect. Ideally, these criteria should be demonstrated across more than one biologically relevant model and subsequently supported by intervention-based human data. Until such evidence is available, KP-associated biomarkers should be described as correlates of disease phenotype or treatment response, not as validated mediators of TB progression.

### 3.12. Therapeutic Implications of KP Modulation

#### 3.12.1. Rationale for Targeting the KP as HDT

The evidence summarized above supports consideration of the KP as a biologically plausible target for HDT in TB [61,62,63]. HDT aims to modulate host immune responses in order to enhance antimicrobial defense while limiting tissue-damaging inflammation [6].

In this context, the KP is relevant because it connects inflammatory signaling, TRP availability, and immunoregulatory metabolite production [14,19,61]. Downstream KYN–AhR signaling provides an additional mechanistic link between KP activation and immune regulation [16,33,58,84]. Activation of this pathway in macrophage, myeloid, and APC contexts has been linked to tolerogenic APC programs, suppressive myeloid phenotypes, and reduced T-cell effector responses [27,61,72]. In TB, increased IDO-related activity has been associated with impaired Th1/Th17-type responses, regulatory immune polarization, altered granuloma organization, and lesion environments that may favor bacterial persistence in selected contexts [61,62,67,68,72].

Taken together, available data support the KP as a biologically plausible, but context-dependent, target for HDT in TB. Across experimental, preclinical, and selected human observational contexts, this pathway has been linked to local immunosuppression, impaired Th1/Th17-type responses, accumulation of suppressive myeloid or regulatory phenotypes, and impaired lesion control or bacterial persistence in some models [27,61,62,67,68,72]. These findings provide a coherent rationale for evaluating context-dependent modulation of the KP as an adjunct to conventional anti-TB treatment [61,62,63,67].

#### 3.12.2. Pharmacological IDO Inhibition

Pharmacological inhibition of IDO-mediated TRP catabolism represents the most direct strategy for therapeutic modulation of the KP in TB. D1-MT has been used experimentally to inhibit IDO-related immunoregulatory activity, although its effects extend beyond direct catalytic inhibition of IDO1, as discussed above. In experimental models, this approach has been associated with reduced IDO-associated local immunosuppression, enhanced selected immune-cell functions, including T-cell and macrophage-associated responses, and improved host control in specific preclinical settings [61,62,63].

Preclinical studies have provided proof-of-concept support for this approach using animal models. In Mtb-infected macaques, pharmacological IDO inhibition was associated with reduced pulmonary bacterial burden and enhanced immune activation [61,62]. In rhesus macaques with Mtb/SIV co-infection receiving cART, addition of D1-MT reduced IDO-related activity and increased pulmonary recruitment, activation, and proliferation of CD4^+^ and CD8^+^ T cells without increasing lung pathology. Granulomas in the D1-MT + cART group also appeared smaller and less necrotic, although this qualitative observation was not formally quantified [63].

Human observational studies and murine or in vitro experimental systems provide complementary support for the biological relevance of IDO-related activity in TB, although they do not establish clinical efficacy of IDO inhibition. In human cohorts, elevated IDO-related activity, increased K/T ratios, and IDO-pathway gene induction have been associated with active disease, greater disease severity, or more severe clinical contexts such as drug-resistant TB [19,27,37,38]. Compartment-specific studies further support local activation of this pathway in inflammatory TB sites. For instance, in severe extrapulmonary manifestations such as TBM, targeted TRP-metabolism profiling has been associated with distinct patient outcomes [35]. Similarly, in tuberculous pleurisy, local pleural fluid contains immunosuppressive factors, including IDO-related activity, that can impair T-cell function and reduce Th1-type responses [47]. Broader metabolic perturbations involving TRP metabolism have also been tracked during conventional first-line anti-TB treatment [43].

Complementary murine and in vitro models provide mechanistic context for these clinical observations. Virulent Mtb strains can induce host metabolic and immunoregulatory pathways that alter local immune responses and tissue environments [67,72]. Mycobacteria-driven activation of the KYN–AhR signaling axis can suppress CXCL9/CXCL10 chemokine responses, providing a mechanism by which KP activation may limit T-cell recruitment to infected tissues [33]. Pathogen-induced IDO/IDO1 expression has also been linked to tolerogenic dendritic-cell programs, including IL-10-producing phenotypes, and to altered macrophage activation pathways [55,72]. Together, these mechanisms may impair Th1-associated immune responses that contribute to mycobacterial control [33,55,72].

These findings support further evaluation of IDO inhibition as an adjunctive host-directed strategy alongside conventional antibiotic therapy. However, translation to human patients requires further clinical studies to define safety, efficacy, timing, and the disease contexts in which IDO inhibition may be beneficial [61,62].

#### 3.12.3. HGF/MET–IDO1 Signaling as an Indirect Therapeutic Axis

MET-dependent signaling represents a potential indirect host-directed therapeutic axis in TB as it appears to positively regulate IDO1 expression in monocytes. HGF stimulation increased IDO1 levels, whereas MET blockade or pharmacologic inhibition reduced it. Pharmacologic IDO inhibition increased GFP-BCG uptake and enhanced CD40, CD83, and CD86 expression, supporting a role for the MET-IDO1 axis in restraining phagocytic and costimulatory activation [60].

These findings are consistent with broader evidence that IDO-related activity can shape monocyte and macrophage activation states during mycobacterial immune responses [55,60,64]. Reduced IDO-related activity or decreased KYN-mediated immunosuppression increases mycobacterial uptake and enhances the expression of immune activation markers [55,60].

Together, these data suggest that MET inhibition may indirectly modulate host immune responses through IDO-related pathways. By enhancing monocyte antimicrobial function and mycobacterial uptake, MET inhibition may support host-directed strategies aimed at improving cellular control of infection. However, all current evidence is limited to non-clinical human experimental systems and does not analyze downstream metabolites of the pathway, such as QUIN or 3-HAA. Further in vivo and translational studies are necessary to confirm the efficacy and safety of MET inhibition in HDT for TB [60].

#### 3.12.4. Potential KP Targets Beyond IDO

Although IDO has received the greatest attention as a pharmacological target in TB, other steps of the KP may also be amenable to therapeutic modulation. Upstream TRP catabolism could potentially be altered through TDO2 inhibition, whereas downstream metabolic branching may be modified by targeting enzymes such as KMO or KATs [8,12]. However, these strategies have not yet been directly evaluated as host-directed interventions in TB. In contrast, the downstream KYN–AhR axis has already been functionally perturbed in experimental Mtb infection: pharmacological AhR inhibition enhanced STAT1 activation, restored CXCL9/CXCL10 production, and increased T-cell chemotaxis in infected macrophage models [33]. Thus, therapeutic modulation of the KP in TB need not be restricted to IDO inhibition and may include upstream enzymes, downstream metabolic branch points, or KYN-dependent signaling, although the evidence supporting these approaches remains substantially less developed than that for IDO interventions.

#### 3.12.5. Other Indirect Modulators of KYN-Related Immunometabolism

##### *Mycobacterium* *vaccae* 

In a murine PTB model, the impact of intragastric *Mycobacterium vaccae* (*M. vaccae*) administration on lung immunoregulatory programs during Mtb infection was studied. Measurements were performed on lung homogenates, and no data on cell-specific IDO expression were shown. In BALB/c mice with TB, bacterial burden and lung expression of immunoregulatory markers, including Foxp3, TGF-β, HO-1, and IDO, were monitored for up to 120 days. Administered one day before Mtb infection, *M. vaccae* reduced pulmonary bacterial burden and reduced pulmonary Foxp3 and TGF-β mRNA, while inducing early increases in HO-1 and IDO mRNA. When treatment was initiated later (from day 32 post-infection), the increase in IDO mRNA was described as minimal but statistically significant [69]. These findings suggest that IDO-related changes may occur as part of broader immunoregulatory remodeling induced by microbial immunomodulation, rather than as a direct consequence of targeted KP inhibition. However, the degree of change was rather moderate.

##### JHU083

In Mtb-infected mice treated with the prodrug of glutamine antagonist JHU083 (6-diazo-5-oxo-L-norleucin), lung metabolomics showed a marked decrease in QUIN, a downstream KP metabolite. Notably, KYN itself was not detectable. Furthermore, direct assessment of the pathway by quantifying IDO1 protein in whole-lung lysates did not reveal changes in IDO1 levels across treatment groups [70]. This suggests that therapeutic benefit may arise through broader remodeling of TRP metabolism rather than through direct suppression of IDO1 itself [70].

#### 3.12.6. Limitations and Translational Considerations

Overall, the current literature supports the KP as a biologically plausible but incompletely validated host-directed therapeutic target in TB, while the evidence remains heterogeneous and largely preclinical. Most human data remain observational or ex vivo, whereas the strongest intervention-based proof-of-concept signals come from animal models.

Importantly, IDO inhibition should not be assumed to be uniformly beneficial across all TB contexts. Experimental evidence indicates that IDO-associated immunoregulation may limit excessive inflammation and tissue damage. However, IDO1 deficiency did not improve bacterial control, pulmonary inflammation, or survival in a murine TB model [14]. Similarly, reduced pulmonary IDO1 expression in mice lacking IFN-γ responsiveness in non-hematopoietic cells was associated with exaggerated IL-17-driven neutrophilic inflammation and reduced survival [68]. However, because IFN-γ unresponsiveness affects multiple pathways, this phenotype cannot be attributed specifically to reduced IDO1 activity. In addition, during infection with the hypervirulent Mtb strain 5186, combined inhibition of IDO and HO-1 increased pulmonary bacillary burden and was accompanied by necrotic lung pathology [67]. It cannot be excluded that IDO-associated regulatory mechanisms may be detrimental in selected inflammatory or high-burden disease settings. Thus, therapeutic targeting of the KP should aim for context-dependent modulation rather than indiscriminate pathway inhibition.

While preclinical data suggest that IDO inhibition may represent a potential host-directed therapeutic strategy for TB, these findings should be interpreted cautiously considering translational experience from other fields. In cancer immunotherapy, selective IDO1 inhibitors such as epacadostat showed promising activity in preclinical models, including effects on tumor-associated immunosuppression and T-cell responses. However, this activity did not translate into clinical benefit in the pivotal Phase 3 ECHO-301/KEYNOTE-252 trial, in which the addition of epacadostat to pembrolizumab did not improve progression-free or overall survival [95,96]. This discrepancy illustrates the limitations of extrapolating therapeutic effects observed in experimental models to complex human immunological settings.

Several factors may contribute to such translational limitations, including the biological redundancy of TRP metabolism. Selective inhibition of IDO1 may be insufficient to suppress pathway activity when alternative enzymes, including TDO and IDO2, or other sources of KYN contribute to maintenance of downstream KYN–AhR signaling [8,97]. Although the tumor microenvironment and the tuberculous granuloma are distinct biological settings, both involve complex and spatially heterogeneous immunoregulatory networks. Thus, successful therapeutic targeting of the KP in TB may require consideration of pathway redundancy, tissue context, and downstream metabolic and signaling activity rather than reliance on inhibition of a single enzyme alone.

Together, these findings indicate that future translational studies should evaluate the pathway at multiple levels simultaneously: direct IDO1 and IDO2 expression, K/T ratio and downstream metabolites or signaling branches such as the KYN-AhR axis, rather than relying on any single parameter as a universal proxy of pathway activity.

Such studies should move beyond association-based findings toward translational and clinical validation, with particular emphasis on intervention-based human studies, careful definition of treatment timing and disease context, and more comprehensive characterization of downstream KP metabolites and signaling networks.

#### 3.12.7. Emerging Technologies and Future Directions

Emerging spatial and single-cell technologies may help resolve the cellular and compartment-specific basis of KP activation in TB. Paired spatial transcriptomics and single-cell RNA sequencing of human TB granulomas have demonstrated that transcriptionally distinct immune and stromal populations occupy defined lesion regions and that gene expression varies according to spatial location [98]. Single-cell multimodal profiling integrating transcriptomic and surface-protein measurements has also identified TB-associated T-cell states that could be distinguished more precisely using combined molecular readouts [99]. More recently, multimodal spatial profiling of non-human primate granulomas identified distinct metabolic microenvironments associated with differences in cellular organization and bacterial burden [100]. In parallel, mass spectrometry imaging has enabled spatial assessment of metabolites in Mtb-infected tissues and revealed metabolic heterogeneity within and between granulomas [101]. Applying these approaches to KP-focused studies could determine whether local IDO1/IDO2 expression, downstream metabolite abundance, and immune-cell states converge within specific granuloma niches, rather than inferring pathway activity primarily from systemic surrogate readouts.

## 4. Conclusions

KP is increasingly recognized as an important regulator of metabolism and immune function in TB. Available evidence indicates that KP activation occurs across multiple cellular and tissue compartments, including myeloid cells, APCs, non-hematopoietic lung compartments, granulomatous lesions, and the CNS. This activation appears to be shaped by IFN-γ signaling, mycobacterial burden, infection duration, strain virulence, and additional host or molecular regulatory mechanisms. 

Functionally, increased IDO-related activity is linked with reduced T-cell proliferation and effector responses, altered chemokine-mediated T-cell recruitment, suppressive myeloid and regulatory T-cell programs, and spatially restricted immunoregulatory niches within granulomas.At the same time, animal models indicate that KP activity is context-dependent and may exert both detrimental and protective effects depending on disease stage, bacterial virulence, host background, and tissue compartment.

Clinically, KP perturbations are evident not only in PTB, but also in pleural TB, TBM and CNS-related manifestations, HIV–TB co-infection, pediatric TB and lymph node TB, supporting the relevance of this pathway across diverse TB phenotypes.

Therapeutically, pharmacologic IDO inhibition and indirect immunometabolic modulators provide preclinical proof-of-concept for targeting this axis, but clinical translation remains insufficiently validated. Future studies should therefore evaluate KP activity using integrated readouts, including IDO 1/2 expression, K/T ratio, downstream metabolites, and KYN–AhR signaling. Prospective clinical trials should determine whether context-specific pathway modulation can safely improve outcomes when combined with conventional anti-TB therapy.

## Figures and Tables

**Table 1:** **Compartment-specific summary of human evidence on the kynurenine pathway in tuberculosis.** Studies are organized according to the investigated population or biological compartment and the KP-related readout or intervention assessed. TB-associated findings and, where available, findings from HIV–TB co-infection are presented separately to distinguish disease-specific and co-infection-related patterns. The biological or clinical implication column summarizes the principal interpretation of each study within the context of KP regulation, immune function, disease phenotype, or biomarker relevance. ↑, increased; ↓, decreased; ↔, no significant or consistent change; -, not assessed or not applicable. 3-HAA, (3-hydroxyanthranilic acid); 3-HK, (3-hydroxykynurenine); AA, (anthranilic acid); AhR, (aryl hydrocarbon receptor); BCG, (Bacillus Calmette–Guérin); CD, (cluster of differentiation); CNS, (central nervous system); CSF, (cerebrospinal fluid); GFP-BCG, (green fluorescent protein-expressing BCG); HGF, (hepatocyte growth factor); HIV, (human immunodeficiency virus); IDO, (indoleamine 2,3-dioxygenase); IDO1, (indoleamine 2,3-dioxygenase 1); IDO2, (indoleamine 2,3-dioxygenase 2); IFIT, i(nterferon-induced protein with tetratricopeptide repeats); IFN-γ, (interferon gamma); K/T ratio, (kynurenine-to-tryptophan ratio); KMO, (kynurenine 3-monooxygenase); KP, (kynurenine pathway); KYN, (kynurenine); KYNA, (kynurenic acid); KYNU, (kynureninase); LTBI, (latent tuberculosis infection); M-MDSCs, (monocytic myeloid-derived suppressor cells); MDR-TB, (multidrug-resistant tuberculosis); MET, (mesenchymal–epithelial transition factor receptor); Mtb, (*Mycobacterium tuberculosis)*; QUIN, (quinolinic acid); TB, (tuberculosis); TBM, (tuberculous meningitis); TDO, (tryptophan 2,3-dioxygenase); Th1, (type 1 T helper cells); Th17, (type 17 T helper cells); TRP, (tryptophan); XA, (xanthurenic acid); *IL23A, (interleukin-23 subunit alpha gene).*

KP-Related Readout	TB	TB-HIV	Biological Implication	Ref.
**ADULTS**				
**Peripheral blood**				
IDO 1/2 soluble IDO	IDO1/soluble IDO ↑; IDO2↓ in one cohort	IDO1 induction reported across HIV cohort; IDO2 induction was more prominent in HIV-positive TB in one study	Systemic TB biomarker; IDO-expressing M-MDSCs associated with greater disease severity	[19,27,28,29]
TRP	↓	↓ vs. HIV+/TB−	Disease-activity or phenotype biomarker	[19,28,30,31,32]
KYN	↑	↔ vs. HIV+/TB−	Disease activity and phenotype biomarker	[28,30,32,33,34]
KYN	TBM: plasma KYN ↓, higher KYN within TBM → higher mortality	-	Prognostic biomarker	[35]
K/T ratio	↑, higher in MDR-TB	↑ vs. HIV+/TB−	Diagnostic, severity, treatment- and vaccine-response biomarker	[19,28,32,34,36,37,38,39,40]
TRP, KYN, K/T ratio	Mood disorders: TRP ↓; KYN and K/T ↑ vs. TB without depressive symptoms	-	Neuropsychiatric phenotype biomarker	[41]
K/T ratio	-	HIV+ LTBI: K/T ↑ vs. HIV− LTBI	Higher K/T associated with reduced circulating Th17 subsets	[42]
QUIN, KYNA	QUIN inversely associated with selected gut taxa/pathways; KYNA positively associated with microbial biosynthetic pathways	-	Gut microbiome-KP association during treatment	[43]
Other KP metabolites	3-HK ↑ in active TB vs. LTBI/controls	AA, KYNA, 3-HAA, XA and QUIN ↓; 3-HK ↔ vs. HIV controls	Potential disease-phenotype marker; biological effects not directly assessed	[30,40]
**Lungs**				
IDO 1/2	↑ in TB lesions and granulomas	-	IDO1-rich niches associated with Treg enrichment and reduced/segregated conventional T-cell infiltration	[18,33,44]
**Pleural fluid**				
TRP	↓	-	Candidate markers distinguishing tuberculous from non-TB pleural effusions	[34,45]
KYN	↑	-	Candidate markers distinguishing tuberculous from non-TB pleural effusions	[34,45]
K/T ratio	↑	-	Candidate markers distinguishing tuberculous from non-TB pleural effusions	[34,45]
Soluble IDO, tissue IDO	↑	-	Associated with local inflammatory/tissue-damage burden	[29,34]
IDO inhibition	Partially restores IFN-γ-T-cell responses	-	IDO-related metabolism contributes to suppression of CD4+ T-cell proliferation and Th1 cytokine responses	[46,47]
**CNS-Brain**				
IDO1/IDO2	Detected in granulomatous and non-granulomatous CNS regions; IDO2 prominent in inferior olive and cerebellum	-	Spatial association with apoptosis in fatal TBM	[48]
3-HAA, QUIN	High levels in granulomatous and non-granulomatous regions	-	Potential contribution to local neurotoxic stress	[48]
**CSF**				
TRP	↓	Higher CSF TRP → higher mortality risk in both HIV− and HIV+ TBM	Prognostic marker	[35,49]
KYN, KYNA, 3-HAA, QUIN	↑	-	Correlate with local inflammation	[35,49]
**Lymph nodes**				
IDO1	↑	↑	IDO1-enriched macrophage/granuloma suppressive phenotype	[50]
**CHILDREN**				
**Peripheral blood**				
soluble IDO	↑	-	Pediatric suppressive-myeloid biomarker	[51]
KYN	↑ in active TB and LTBI	-	Ex vivo pediatric biomarker	[52]
K/T ratio	↑ in active TB and LTBI	-	Ex vivo pediatric biomarker	[52]
TRP	↓	-	Pediatric biomarker	[53]
**HUMAN STUDIES IN VITRO, EX VIVO**				
**Macrophages and macrophage-like cells**				
IDO 1/2, TDO2; KMO, KYNU	Mtb infection ↑ IDO1/TDO2, KMO, KYNU; IDO2 ↔; Rv1737c ↑ IDO1; IDO remained low after vitamin-D polarization; H37Rv induced stronger IDO1/TDO2 than H37Ra/BCG; IFIT overexpression ↑ IDO1, whereas IFIT knockdown ↓ IDO1	IDO1 and KYNU ↑ in Mtb-infected and HIV/Mtb-co-infected macrophages	Context-dependent KP induction; vitamin D-associated low IDO coincides with improved intracellular Mtb control; AhR promotes IL1B/IL23A expression	[14,54,55,56,57,58,59]
QUIN	↑	-	IFN-γ biases macrophage KP output toward QUIN rather than PIC; functional consequence of this shift not tested	[14]
PIC	↔	-	IFN-γ biases macrophage KP output toward QUIN rather than PIC; functional consequence of this shift not tested	[14]
KYN–AhR	Activated	-	T-cell suppression	[33]
**Monocytes**				
HGF/MET–IDO1; IDO inhibition	HGF/MET ↑ IDO1; MET blockade or IDO inhibition ↑ GFP-BCG uptake	-	IDO-related signaling restrains mycobacterial uptake and monocyte co-stimulatory activation	[60]
IDO inhibition, addition of 3-OH-kynurenine	IDO inhibition → ↑ CD4+/CD8+ T-cell proliferation; 3-OH-kynurenine reversed this effect	-	T-cell suppression	[30]

**Table 2:** **Compartment-specific summary of animal and animal-derived experimental evidence on the kynurenine pathway in tuberculosis.** Studies are organized according to animal species, biological compartment, or experimental model and the KP-related parameter or intervention assessed. TB/Mtb-associated patterns are presented separately from findings obtained during co-infection or experimental intervention. The biological implication column provides an integrated interpretation of the principal findings, including their relevance to KP regulation, immune responses, bacterial control, and disease pathology. ↑, increased; ↓, decreased; ↔, no significant or clear change; -, not assessed or not applicable. 1-MT-DL, (1-methyl-DL-tryptophan); 3-HAA, (3-hydroxyanthranilic acid); 3-HK, (3-hydroxykynurenine); Acr1, (alpha-crystallin 1); AhR, (aryl hydrocarbon receptor); APC, (antigen-presenting cell); ART, (antiretroviral therapy); BAL, (bronchoalveolar lavage); cART, (combination antiretroviral therapy); CD4, (cluster of differentiation 4); CFU, (colony-forming units); CXCL9, (C-X-C motif chemokine ligand 9); CXCL10, (C-X-C motif chemokine ligand 10); D1-MT, (D-1-methyltryptophan); DC, (dendritic cell); IDO, (indoleamine 2,3-dioxygenase); IDO1, (indoleamine 2,3-dioxygenase 1); IDO2, (indoleamine 2,3-dioxygenase 2); iBALT, (inducible bronchus-associated lymphoid tissue); K/T ratio, (kynurenine-to-tryptophan ratio); KP, (kynurenine pathway); KYN, (kynurenine); LTBI, (latent tuberculosis infection); ME, (moxifloxacin and ethambutol); mRNA, (messenger RNA); Mtb, (*Mycobacterium tuberculosis)*; PMN-MDSCs, (polymorphonuclear myeloid-derived suppressor cells); QUIN, (quinolinic acid); SIV, (simian immunodeficiency virus); TB, (tuberculosis); Th17, (type 17 T helper cells); TRP, (tryptophan); ZnPp, (zinc protoporphyrin IX); *IL23A, (interleukin-23 subunit alpha gene).*

KP-Related Readout	TB	Co-Infection/Intervention	Biological Implication	Ref.
**MACAQUES**				
**PERIPHERAL BLOOD**				
KYN, TRP, K/T ratio	K/T ↔ before intervention	D1-MT → >95% ↓ K/T	IDO-related activity is pharmacologically suppressible in vivo	[61]
**BAL/AIRWAY**				
KYN/K/T ratio	KYN^+^ BAL cells ↑; K/T ↑ in Mtb/SIV	D1-MT ± ME/cART → ↓ KYN-related readouts	Reduced airway KP activity accompanies improved immune or microbiological control	[61,62,63]
**LUNG/PULMONARY TISSUE**				
IDO	↑ in active TB vs. LTBI; correlates with Mtb burden	Chemotherapy → ↓ IDO	Pulmonary IDO tracks disease burden	[61]
IDO1	IDO1^+^ myeloid cells enriched in iBALT during Mtb/SIV reactivation	ART restores blood/BAL CD4^+^ cells but not lung-interstitial recovery	Local IDO1 may contribute to compartment-specific immune dysfunction	[64]
**GRANULOMA/LESION**				
IDO/IDO1; IDO1-high PMN-MDSCs	Enriched in myeloid granuloma regions	D1-MT-based interventions → ↑ T-cell access and improved lesion control	Granuloma IDO1 is associated with spatial immune restriction	[61,62,63,65,66]
**MICE—IN VIVO**				
**PERIPHERAL BLOOD/SERUM**				
IDO1/IDO2	IDO1 ↑; IDO2 ↓ during early infection	-	Isoform-specific regulation during infection	[28]
KYN/TRP	Serum KYN ↑ at 3 weeks in one model; at 8 weeks in another H37Rv model, TRP ↓ and KYN ↓	KYN or myeloid AhR manipulation altered CXCL9/10 and T-cell recruitment	Systemic KYN responses are model-dependent; KYN–AhR can impair T-cell recruitment	[31,33]
**LUNG/PULMONARY TISSUE**				
IDO/IDO1	Pulmonary IDO1 ↑; magnitude depends on strain/stage and burden	IDO1−/− → no change in burden/survival; oral *M. vaccae* or late 1-MT-DL → ↓ CFU; 1-MT-DL + ZnPp in hypervirulent TB → ↑ CFU and necrosis	Functional effects of IDO-related modulation depend on infection stage and strain.	[14,30,33,61,67,68,69]
KYN–AhR	Mtb → ↑ IDO1/KYN–AhR signaling	KYN worsened T-cell recruitment and burden; Ahr loss had opposite effects	KYN–AhR can impair protective pulmonary immunity	[33]
QUIN	-	JHU083 → ↓ QUIN, burden and pathology; ↑ survival	QUIN reduction accompanies benefit, but causality is unresolved	[70]
**MICE — IN VITRO/EX VIVO**				
**DENDRITIC CELLS/MACROPHAGES**				
IDO/IDO1	Mtb/Acr1 → ↑ IDO1; H37Rv induced tolerogenic DC phenotype	IDO1 loss/1-MT-DL → ↑ CD4^+^ proliferation; no improved macrophage Mtb control	APC IDO1 suppresses T-cell responses but is not required for intracellular Mtb control	[14,61,71,72]
**T-CELL DIFFERENTIATION**				
KYN/3-HK/3-HAA	-	Exogenous metabolites → ↓ Th17 differentiation; 3-HAA has the strongest potential	KP metabolites directly suppress Th17 responses	[68]
**MACROPHAGE SIGNALING**				
KYN–AhR signaling	Mtb activates IDO1–KYN–AhR signaling	AhR loss/inhibition altered CXCL9/10 and Il23a responses	AhR effects are context-dependent	[33,58]
**OTHER SPECIES**				
**ZEBRAFISH—WHOLE ORGANISM**				
TRP/KYN	*M. marinum* → TRP ↓ without KYN ↑	-	TRP depletion does not necessarily indicate increased TRP-to-KYN conversion	[31]

## Data Availability

No new data were created or analyzed in this study. Data sharing is not applicable to this article.

## Abbreviations

The following abbreviations are used in this manuscript:

1,25(OH)_2_D_3_	1,25-dihydroxyvitamin D_3_
3-HAA	3-hydroxyanthranilic acid
AA	anthranilic acid
AhR	aryl hydrocarbon receptor
APCs	antigen-presenting cells
ART	antiretroviral therapy
BAL	bronchoalveolar lavage
BBB	blood–brain barrier
BCG	Bacillus Calmette–Guérin
BMDCs	bone marrow-derived dendritic cells
cART	combination antiretroviral therapy
CCR5	C-C chemokine receptor type 5
CD	cluster of differentiation
CFSE	carboxyfluorescein succinimidyl ester
CFU	colony-forming units
CNS	central nervous system
COX-2	cyclooxygenase-2
CSF	cerebrospinal fluid
CTLA-4	cytotoxic T-lymphocyte-associated protein 4
CXCL9	C-X-C motif chemokine ligand 9
CXCL10	C-X-C motif chemokine ligand 10
CXCR3	C-X-C motif chemokine receptor 3
D1-MT	D-1-methyltryptophan
1-MT	1-methyltryptophan
1-MT-DL	1-methyl-DL-tryptophan
DCs	dendritic cells
DNA	deoxyribonucleic acid
GFP	green fluorescent protein
GFP-BCG	green fluorescent protein-expressing Bacillus Calmette–Guérin
GM-CSF	granulocyte–macrophage colony-stimulating factor
HDT	host-directed therapy
HGF	hepatocyte growth factor
HIV	human immunodeficiency virus
HO-1	heme oxygenase-1
IDO	indoleamine 2,3-dioxygenase
IDO1	indoleamine 2,3-dioxygenase 1
IDO2	indoleamine 2,3-dioxygenase 2
IFITs	interferon-induced proteins with tetratricopeptide repeats
IFN-γ	interferon-γ
IL-1β	interleukin-1 beta
IL-4	interleukin-4
IL-10	interleukin-10
IL-17	interleukin-17
IL-22	interleukin-22
IL-23	interleukin-23
*IL1B*	*interleukin 1 beta gene*
*IL23A*	*interleukin 23 subunit alpha gene*
JAK	Janus kinase
K/T ratio	kynurenine-to-tryptophan ratio
KATs	kynurenine aminotransferases
Ki-67	marker of proliferation Ki-67
KMO	kynurenine 3-monooxygenase
KP	kynurenine pathway
KYN	kynurenine
KYNA	kynurenic acid
*LAG-3*	*lymphocyte activation gene 3*
LC–MS/MS	liquid chromatography–tandem mass spectrometry
LDH	lactate dehydrogenase
LPS	lipopolysaccharide
LTBI	latent tuberculosis infection
MET	MET receptor tyrosine kinase
M-MDSCs	monocytic myeloid-derived suppressor cells
mRNA	messenger ribonucleic acid
Mtb	*Mycobacterium tuberculosis*
MVA85A	modified vaccinia Ankara virus expressing antigen 85A
NFAT	nuclear factor of activated T cells
NR4A1	nuclear receptor subfamily 4 group A member 1
PBMCs	peripheral blood mononuclear cells
PD-1	programmed cell death protein 1
PD-L1	programmed death-ligand 1
PHQ-9	Patient Health Questionnaire-9
PIC	picolinic acid
PLWH	people living with HIV
PMN-MDSCs	polymorphonuclear myeloid-derived suppressor cells
PRISMA	Preferred Reporting Items for Systematic Reviews and Meta-Analyses
PTB	pulmonary TB
QUIN	quinolinic acid
RNA	ribonucleic acid
SIV	simian immunodeficiency virus
SNP	single-nucleotide polymorphism
SOCS3	suppressor of cytokine signaling 3
STAT1	signal transducer and activator of transcription 1
TB	tuberculosis
TBM	tuberculous meningitis
TDO	tryptophan 2,3-dioxygenase
TDO2	tryptophan 2,3-dioxygenase 2
TGF-β	transforming growth factor-β
THP-1	human monocytic leukemia cell line
Th1	type 1 T helper cells
Th2	type 2 T helper cells
Th17	type 17 T helper cells
TIM-3	T-cell immunoglobulin and mucin-domain-containing protein 3
TNF-α	tumor necrosis factor-α
Treg	regulatory T cell
TRP	tryptophan
